# Complete *CSN1S2* Characterization, Novel Allele Identification and Association With Milk Fatty Acid Composition in River Buffalo

**DOI:** 10.3389/fgene.2020.622494

**Published:** 2021-02-04

**Authors:** Gianfranco Cosenza, Daniela Gallo, Barbara Auzino, Giustino Gaspa, Alfredo Pauciullo

**Affiliations:** ^1^Department of Agriculture, University of Napoli Federico II, Portici, Italy; ^2^Department of Agricultural, Forest and Food Sciences, University of Torino, Grugliasco, Italy

**Keywords:** *CSN1S2*, alleles, candidate gene, mediterranean river buffalo, milk, palmitic acid

## Abstract

The αs2-casein is one of the phosphoproteins secreted in all ruminants' milk, and it is the most hydrophilic of all caseins. However, this important gene (*CSN1S2*) has not been characterized in detail in buffaloes with only two alleles detected (reported as alleles A and B), and no association studies with milk traits have been carried out unlike what has been achieved for other species of ruminants. In this study, we sequenced the whole gene of two Mediterranean river buffalo homozygotes for the presence/absence of the nucleotide C (g.7539G>C) realized at the donor splice site of exon 7 and, therefore, responsible for the skipping of the same exon at mRNA level (allele B). A high genetic variability was found all over the two sequenced *CSN1S2* alleles. In particular, 74 polymorphic sites were found in introns, six in the promoter, and three SNPs in the coding region (g.11072C>T, g.12803A>T, and g.14067A>G) with two of them responsible for amino acid replacements. Considering this genetic diversity, those found in the database and the SNP at the donor splice site of exon 7, it is possible to deduce at least eight different alleles (*CSN1S2* A, B, B1, B2, C, D, E, and F) responsible for seven different possible translations of the buffalo αs2-casein. Haplotype data analysis suggests an evolutionary pathway of buffalo *CSN1S2* gene consistent with our proposal that the published allele *CSN1S2* A is the ancestral αs2-CN form, and the B2 probably arises from interallelic recombination (single crossing) between the alleles D and B (or B1). The allele *CSN1S2* C is of new identification, while *CSN1S2* B, B1, and B2 are deleted alleles because all are characterized by the mutation g.7539G>C. Two SNPs (g.7539G>C and g.14067A>G) were genotyped in 747 Italian buffaloes, and major alleles had a relative frequency of 0.83 and 0.51, respectively. An association study between these SNPs and milk traits including fatty acid composition was carried out. The SNP g.14067A>G showed a significant association (*P* < 0.05) on the content of palmitic acid in buffalo milk, thus suggesting its use in marker-assisted selection programs aiming for the improvement of buffalo milk fatty acid composition.

## Introduction

The αs2-casein (207 aa) is one of the phosphoproteins (αs1, β, αs2, and k) secreted in ruminants' milk in the form of stable calcium–phosphate micelles, and it is the most hydrophilic of all caseins. The αs2-casein (αs2-CN) appears to be readily susceptible to proteolysis as assessed by the activities of chymosin and plasmin toward the protein. The molecular weight of this protein was assessed to be 22,741 Da in buffalo vs. 25,226 in cattle (Feligini et al., 2009).

The proportion of αs2-CN in milk changes considerably between species and is absent from human and marsupial milk (Kim et al., 2015). In buffalo milk, the αs2-CN is the third most abundant casein fraction (4.99 g/L), and the corresponding coding gene (*CSN1S2*) showed a lower translation efficiency (0.25) compared to the other casein genes as *CSN3* (k-CN, 2.69), *CSN2* (β-CN, 2.39), and *CSN1S1* (αs1-CN, 1.31) (Cosenza et al., 2011).

Among ruminants, goat and sheep showed a higher level of genetic diversity at *CSN1S2*, and nowadays, at least seven alleles associated with three different αs2-CN levels have been characterized in both species (Boisnard et al., 1991; Ramunno et al., 2001a,b; Giambra and Erhardt, 2011). In cattle, only four variants A, B, C, and D have been found (Farrell et al., 2004). The alleles B and C are specific for the zebu and yak cattle, respectively (Ibeagha-Awemu et al., 2007).

Conversely, this *locus* is less polymorphic in buffalo, probably as a result of the little studies realized in this species. Chianese et al. (1996) have reported three variants that differ for the content of phosphates, and D'Ambrosio et al. (2008) have indicated different αs2-CN isoforms with 13, 12, 11, and 10 phosphate groups realized at the same positions as those observed in cattle. At the DNA level, the only example of biallelic polymorphism (alleles A and B) observed, so far, at the buffalo *CSN1S2* has been identified and characterized by Cosenza et al. (2009a). The mutation that characterizes the allele B is an SNP (FM865620:g.773G>C) realized at the donor splice site of exon 7 and, therefore, responsible for the skipping of the same exon at mRNA level.

Contrary to what has been studied in other ruminants, until now, this important gene has not been characterized in detail in buffaloes. In 2006, Sukla et al. characterized the cDNA sequence in the Murrah breed (GeneBank no. DQ173244.1), and only very recently, the complete and annotated sequence of *CSN1S2* gene has been published for the Mediterranean breed (*Bubalus bubalis* breed Mediterranean chromosome 7, ASM312139v1, whole genome shotgun sequence; GenBank no. NC_037551.1, 32020000-32040337, complement).

Although a new reference genome assembly (UOA_WB_1) has been published (Low et al., 2019), and the first SNP array designed specifically for buffaloes has become available (Iamartino et al., 2017), its use is still very limited. Therefore, the candidate gene approach is still today a valid method for the identification of genetic variability and its relationship with milk production traits. Several studies have been carried out in river buffalo aiming the discovery of polymorphisms in *loci* coding for milk proteins that, in other ruminants, have well-known effects on milk characteristics (Masina et al., 2007; Cosenza et al., 2009a,b; Balteanu et al., 2013; Vinesh et al., 2013; Cosenza et al., 2015). For instance, these studies allowed the identification of positive associations between markers at *CSN1S1* and *CSN3* and traits of economic interest, like the protein yield (Cosenza et al., 2015) and milk coagulation properties (Bonfatti et al., 2012a,b). Conversely, in this respect, no association studies have been carried out in the buffalo for the *CSN1S2* so far, unlike what has been achieved for other species of ruminants. In fact, significant differences were found between genotypes of the goat, sheep, and cattle *CSN1S2 locus* in relation to milk protein and casein content (Ramunno et al., 2001b; Noce et al., 2016; Ardicli et al., 2018). Besides, *CSN1S2* genotypes were significantly associated with milk and/or fat yield in goat and sheep (Wessels et al., 2004; Lan et al., 2005; Yue et al., 2013; Vacca et al., 2018). For years, the interest of several research groups also focused on the study of connection between milk fat and fatty acid composition and the different milk protein polymorphisms and/or genetic polymorphisms of casein-encoding genes (Bobe et al., 1999, 2004; Chilliard et al., 2006; Cebo et al., 2012). In particular, it has been shown that fat globule size, the incidence of each globule size class on total measured bovine milk fat globules, and fatty acid composition are strongly influenced by single casein *loci* or casein haplotype (Perna et al., 2016).

The aim of this study was to sequence the whole *CSN1S2* for the samples reported as alleles A and B by Cosenza et al. (2009a), to characterize and annotate extensively the gene, to compare the alleles in their complex genetic diversity, and to investigate possible association with traits that might affect the nutritional and technological quality of buffalo milk.

## Materials and Methods

### DNA Samples and Phenotypes Collection

Samples used in this study belong to DNA collections of the University of Napoli Federico II and University of Turin.

The original biological tissue used for DNA isolation was blood, collected during routine treatments according to Italian national rules on animal welfare and achieved by official veterinarians in collaboration with the Italian National Association of Buffalo Breeders (A.N.A.S.B.).

DNA was isolated from leukocytes using the procedure described by Goossens and Kan (1981). DNA concentration and the OD_260/280_ ratio of the samples were measured by a Nanodrop ND-2000 Spectrophotometer (Thermo Fisher Scientific Inc., Waltham, MA, USA).

DNA from two Mediterranean river buffaloes, homozygotes for the alleles A (FM865620:g.773G) and B (FM865621:g.773C) as determined by Cosenza et al. (2009a), have been used for the complete sequencing of the *CSN1S2*. In addition, individual DNA samples randomly chosen from 747 female Mediterranean river buffaloes belonging to 14 farms with intensive breeding system, located in Salerno, Caserta, and Potenza provinces (Southern Italy) were used for population analysis.

For assessing possible associations between polymorphisms identified at the *CSN1S2 locus* and milk traits, such as milk yield, fat percentage, single fatty acid percentage, and fatty acid classes, we used single milk samples collected from a sub-group of 310 lactating buffaloes. These subjects were at third calving, had similar days in milking (DIM: 110–120), feeding management and diet, with a reduced occurrence of unsaturated fatty acids, compared to graze-based systems.

Fatty acid (FA) composition, FA classes, and fat percentage of the 310 individual milk samples have been assessed and previously reported by Cosenza et al. (2017a, 2018a). The same phenotypes were also used in the present work to assess possible associations with the genetic diversity found at the *CSN1S2 locus* by using the mixed linear model as reported by Cosenza et al. (2017a).

### PCR Amplification Conditions and Genotyping

Using primers designed on bubaline genome (GenBank accession no. NC_037551.1, from 32020000 to 32040337 complement) and bubaline mRNA sequence (GenBank accession nos. FM865618.1, FM865619.1) (Supplementary Table 1), the DNA regions of the *CSN1S2* gene spanning from the 5′- to the 3′-UTR of two Mediterranean river buffalo homozygotes for the alleles A and B were amplified by iCycler (BioRad, CA, USA). A typical 50-μl PCR reaction mix including 100 ng of genomic DNA, 50 mM KCl, 10 mM Tris-HCl (pH 9.0), 0.1% Triton X-100, 3 mM MgCl_2_, 200 nmol of each primer, dNTPs each at 400 μM, 2.5 U of Taq DNA Polymerase (Promega, Madison, WI), and 0.04% BSA. The thermal condition for the amplification consisted of an initial denaturation at 95°C for 4 min, followed by 35 cycles at 94°C for 45 s, 54.0–57.4°C for 45 s (according to the amplicon) and 72°C for 2 min. A final extension of 10 min was accomplished to end the reaction. All PCR products were analyzed directly by electrophoresis in 1.5% TBE agarose gel (Bio-Rad, CA, USA) in 0.5X TBE buffer and stained with SYBR® green nucleic acid stain (Lonza Rockland, Inc., USA). PCR products were sequenced on both strands at CEINGE–Biotecnologie Avanzate (Naples, Italy) using Sanger DNA sequencing technology.

The entire panel of 747 Mediterranean river buffalo DNA samples was genotyped in outsourcing (KBiosciences, Herts, UK, http://www.kbioscience.co.uk) for the SNPs g.7539G>C (FM865620:g.773G>C) and g.14067A>G.

### Bioinformatics and Statistical Analysis

Allelic frequencies and Hardy–Weinberg equilibrium (chi square test) were calculated. Homology searches, comparisons among nucleotide and amino acid sequences, and multiple alignments for polymorphism discovery were accomplished using Dnasis Pro (Hitachi Software Engineering Co.). Measures of linkage disequilibrium (D' and *r*^2^) were estimated using Haploview software ver. 4.2 (http://www.broadinstitute.org/haploview/haploview). The haplotype structure was defined according to Gabriel et al. (2002). The regulatory regions were analyzed for potential transcription factors (TFs) by Transfac® 7.0. (http://gene-regulation.com/index2.html). Associations between *CSN1S2* genotypes and fat traits were tested using a mixed linear model by SAS (*ver* 9.2) as reported by Cosenza et al. (2017a).

## Results

### *CSN1S2* Gene Structure in Mediterranean River Buffalo

By using genomic DNA as template, we sequenced the whole gene encoding the αs2-casein (*CSN1S2*) of two Mediterranean river buffalo homozygotes for the presence/absence of the nucleotide C (FM865620:g.773G>C) that caused inactivation of the intron 7 splice donor site and, consequently, the allele-specific exon skipping characteristic of the *CSN1S2* B allele (GenBank accession nos. MW159135 and MW159136).

Using as reference the sample homozygote for the allele FM865620:g.773G (previously misidentified as *CSN1S2* A and from now named allele *CSN1S2* D), the sequenced DNA region including the *CSN1S2* gene is about 20,300-bp long, and it includes 1,025 bp of exonic regions, 17,578 bp of intronic regions, 937 nucleotides at the 5′ flanking region, and 707 nucleotides at the 3′ flanking region. The level of sequence similarity with the allele *CSN1S2* B is about 98% as a consequence of an elevated polymorphism.

The main feature of the buffalo *CSN1S2* gene is the extremely split architecture. It contains 18 exons ranging in size from 21 (exon 4) to 267 bp (exon 18). The first exon (44 bp) is not coding at all. The whole highly conserved signal peptide (15 amino acids, MKFFIFTCLLAVALA) of the mature protein (207 amino acids) is encoded by the nucleotides 13–57 of exon 2 (63 bp), and the translation stop codon TAA is created by nucleotides 10–12 of exon 17. The deduced CDS length of bubaline *CSN1S2* gene is 669-bp long. These results are in agreement with what was reported by Sukla et al. (2006). All splice junctions follow the 5′ GT/3′ AG splice rule, similarly as it was described in different ruminant species. The only peculiarity is represented by the polymorphism at the splice donor site of exon 7 of the allele *CSN1S2* B (g.7539G>C, corresponding to FM865620:g.773G>C).

Consequently, the *CSN1S2* B allele (GenBank MW159136) compared to the *CSN1S2* D (GenBank MW159135) allele is characterized by 17 exons.

Finally, different microsatellite sequences are present in the buffalo *CSN1S2* gene, many of which flanking retroposonic sequences (Supplementary Figure 1).

### Polymorphism Detection

The analysis and the alignment of the *CSN1S2* intronic sequences of the two subjects used in this study have highlighted a remarkable genetic diversity.

In detail, 74 polymorphic sites (24 transversions, 37 transitions, 13 deletions/insertions) and several variable microsatellites were found between the two sequenced subjects (Supplementary Figure 1, Supplementary Table 2). Except for the g.7539G>C at the splicing donor site of exon 7 and causative event of the *CSN1S2* B allele, none of the remaining polymorphisms are apparently located in the regulatory regions (splicing donor/acceptor site, enhancer/silencer, etc.) and as a consequence, we hypothesize that they do not affect the *CSN1S2* expression.

Then, the comparison between our sequences and the reference sequence recorded in GenBank (NC_037551.1) highlighted further 15 new intronic mutations. In particular, two polymorphisms are responsible for the differences in the number of mononucleotide thymine (T) repeats, while one is a multiple substitution: NC_037551.1:g.32034006A>G>T (Supplementary Table 2). This genomic sequence is particularly interesting because it is also characterized by a cytosine at the splice donor site of exon 7 (NC_037551.1:g.32033131C), and consequently, it can be considered an allele B derived.

As expected, the comparison of the exonic regions showed a reduced level of polymorphism. We identified three SNPs in total. The first, g.11072C>T, is located at the 18th nucleotide of exon 13; it is a conservative SNP, and it is not generating any amino acid change. The further SNPs are located at the 16th nucleotide of exon 14 and at the 31st nucleotide of exon 16. They are the transversions g.12803A>T and g.14067A>G responsible for the amino acid substitutions p.I162>F and p.190T>A, respectively (Supplementary Figure 1). The g.14067A>G has been observed in heterozygosis in the sample homozygote for the SNP g.7539G, giving two new alleles named *CSN1S2* C (g.14067A) and D (g.14067G).

Furthermore, by comparing the sequences analyzed in this work and those available in the database (https://blast.ncbi.nlm.nih.gov/Blast.cgi) for the buffalo *CSN1S2* gene, it is possible to identify other four exonic polymorphisms (Table 1) and consequently several haplotypes.

**Table 1:** Discovery and diffusion of the genetic variants of buffalo αs2-casein-encoding gene (*CSN1S2*).

***CSN1S2* alleles**	**Exon, nucleotide, and amino acid position**																**Breed**
	**Exon 2**		**Exon 7**		**Exon 11**		**Exon 11**		**Exon 13**		**Exon 14**		**Exon 16**		**Exon 16**		
	**nt**	**aa**	**nt**	**aa**	**nt**	**aa**	**nt**	**aa**	**nt**	**aa**	**nt**	**aa**	**nt**	**aa**	**nt**	**aa**	
	**3165**	**5**	**7539**	**58-66**	**9220**	**127**	**9221**	**128**	**11072**	**153**	**12803**	**162**	**14067**	**190**	**14141**	**214**	
A^1^	T	I	G	EVIRNANEE	T	V	A	K	C	T	T	F	G	A	C	N	Murrah
C^2^	T	I	G	EVIRNANEE	T	V	A	K	C	T	A	I	G	A	C	N	Mediterranean
D^3^	T	I	G	EVIRNANEE	T	V	A	K	C	T	A	I	A	T	C	N	Mediterranean/ Egyptian/ Murrah
E^4^	C	I	G	EVIRNANEE	T	V	A	K	C	T	A	I	A	T	G	K	Murrah
F^5^	T	I	G	EVIRNANEE	A	V	G	E	C	T	A	I	A	T	C	N	Murrah
B^6^	T	I	C	—	T	V^117^	A	K^118^	T	T^144^	T	F^153^	G	A^181^	C	N^205^	Mediterranean
B1^7^	T	I	C	—	T	V^117^	A	K^118^	C	T^144^	T	F^153^	G	A^181^	C	N^205^	Carabao
B2^8^	T	I	C	—	T	V^117^	A	K^118^	C	T^144^	A	I^153^	A	T^181^	C	N ^205^	Mediterranean

*The CSN1S2 A allele is the putative original one from which the different alleles originated*.

*Numbering refers to CSN1S2 allele D (GenBank MW159135) both for nucleotides (nt) and the corresponding predicted protein (aa)*.

*References—**1**: NM_001290865.1; **2**: present work; **3**: XM_006071123.2, KY399458.2, FM865618.1, JQ292811.1, AJ005431.2, present work (MW159135); **4**: DQ173244.1; **5**: DQ133467.1; **6**: FM865619.1; present work (MW159136); **7**: KX896650.1; **8**: NC_037551.1*.

Two SNPs were conservative, the transition g.3165T>C (27th nt of the exon 2, GenBank acc. no. DQ173244.1) and the transversion g.9220T>A (90th nt of the exon 11, GenBank acc. no. DQ133467.1). The other SNPs were not conservative: the transition g.9221A>G (91st nt of the exon 11, GenBank acc. no. DQ133467.1), responsible for the amino acid change p.128K>E and the transversion g.14141C>G (105th nt of the exon 16, GenBank acc. no. DQ173244.1), which generates the amino acid replacement p.214N>K.

### Rearrangement of Allele Nomenclature and Phylogenetic Relationship Among the Markers

Considering all the SNPs (both from the database and newly determined in the present study), it is possible to deduce at least eight different alleles (*CSN1S2* A, B, B1, B2, C, D, E, and F; Table 1) responsible for seven different possible translations of the buffalo αs2-casein.

**Figure 1:**
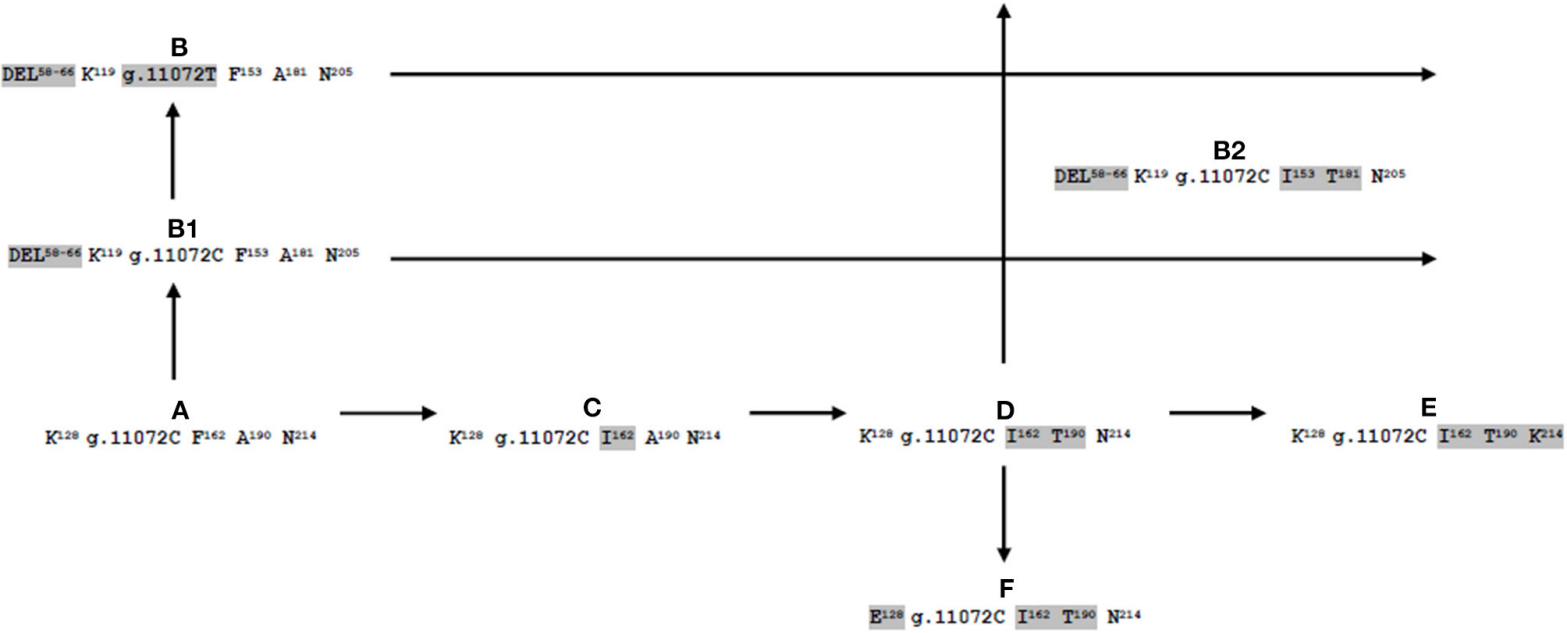
Possible evolutionary pathway of buffalo αs2-casein-encoding gene (*CSN1S2*).

The allele that we named *CSN1S2* A (GenBank NM_001290865) is stated as ancestral αs2-CN form according to nucleotide and amino acid sequence of cattle and goat. By several mutational events often responsible for either amino acid substitution or deletions, starting from *CSN1S2* A, we propose two different phylogenetic road maps. The first map generates four alleles that are different for a single amino acid substitution: p.162F>I (*CSN1S2* C, present work), p.162F>I and p.190A>T (*CSN1S2* D, XM_006071123.2, KY399458.2, FM865618.1, JQ292811.1, AJ005431.2, present work), p.162F>I, p.190A>T, and p.214N>K (*CSN1S2* E, DQ173244.1). Similar to *CSN1S2* E, also the allele named *CSN1S2* F (DQ133467.1) originated from the allele *CSN1S2* D because they differ from each other only for the amino acid substitution p.128K>E (Figure 1).

The second phylogenetic road map is generated by the point mutation g.7539G>C, which brings to the inactivation of the intron 7 splice donor site. Thus, as a consequence, the alleles named *CSN1S2* B1 (KX896650) and B (FM865619.1; present work) are characterized by the complete skipping of exon 7 (nine amino acids, EVIRNANEE from 58 to 66). Moreover, *CSN1S2* B1 and B differ from each other for the single polymorphism g.11072C>T in the exon 13 (Table 1, Figure 1).

Finally, the comparison of specific haplotypes defined for each of the *CSN1S2* alleles (Table 1) indicates that the B2 probably arises from interallelic recombination (single crossing) between alleles D and B or B1 (Figure 1).

### Regulatory Elements and Polymorphism Detection at the Gene Promoter

Variations in regulatory regions are known to affect the composition, structure, and expression of milk caseins (Martin et al., 2002; Szymanowska et al., 2003; Cosenza et al., 2007, 2016). Therefore, the proximal promoter regions of both *CSN1S2* D and B alleles were sequenced and characterized.

Using the database Transfact® 7.0, we identified the potential transcription factors (TFs) that could affect the gene expression. Together with the TATA box, we identified the following TFs: C/EBP (CCAAT/enhancer-binding protein), Oct-1 (octamer-binding factor-1), HNF-3beta (hepatocyte nuclear factor-3 beta), AP (activator protein), YY1 (Yiang Yang factor-1), POU1F1a (Pit1, growth hormone factor 1), PR (progesterone receptor), GR (glucocorticoid receptor), and MGF (mammary gland factor) (Supplementary Figure 1). This gene structure is similar to the homologous gene identified in *Bos taurus* and *Bos indicus* (Kishore et al., 2013) as demonstrated by the conserved position of the TATA box (between nucleotide −25 and −30, where +1 is the first nucleotide of the first exon).

The sequence comparison of the gene promoters for the alleles *CSN1S2* B and D showed six SNPs in total: four transitions, one transversion, and the deletion of one adenine (Supplementary Figure 1, Supplementary Table 2). None of the polymorphisms identified generates or deletes known TFs, and consequently, no influence on gene expression was expected.

However, in the comparison with the only *Bubalus bubalis* promoter sequence available at GenBank (accession number EF066480), three additional sites of variation were detected: g.595A>G, g.620_622delG, and g.996T>A. The latter polymorphism fell within the putative transcriptional factor binding site for Oct-1.

At the 3′-end of the gene, the polyadenylation site AATAAAA is located between nucleotides 247–253, with reference to the first nucleotide of the 18th exon. In addition, a G/T cluster was found downstream of the poly-A site. This sequence motif also contributes to the information for the polyadenylation. Both the AATAAA sequence and the G/T cluster are underlined in Supplementary Figure 1. With the exception of a polymorphic stretch of T, we do not report any further mutation in this region.

### Repeated Sequences Within the Mediterranean River Buffalo *CSN1S2* Gene

The buffalo *CSN1S2* gene sequence is characterized by at least 13 retrotransposons (Supplementary Figure 1). In particular, the first (A) is located in the promoter region (GenBank MW159135 from 136 to 305) and appears to be a retroposon of Bov-tA2 type.

Further, two elements are located in the first intron (B, from 1,122 to 1,290, and C, from 1,952 to 2,137, respectively) and showed a strong similarity with an L1_Art sequence. Then, we found two Bov-tA2 located in intron 2 that we named element D (from the nucleotide 3,696 to 3,906) and element E (from nucleotide 4,155 to 4,313). At intron 8, we found a Bov-B (element F, from nucleotide 8,320 to 8,574), whereas in intron 12, we identified a Bov-A2 (retroposon G, from nucleotide 10,338 to nucleotide 10,621). Furthermore, five retroposons (H, I, L, M, and N) are located in intron 13 (Bov-tA1, from 12,138 to 12,356), in intron 15 (Bov-A2, from 13,313 to 13,587), and in the intron 17 (Bov-tA1, from 15,051 to 15,236, Bov-B from 15,931 to 17,485, and Bov-A2, from 17,763 to 18,046). Finally, a further element Bov-tA2 (O) is located in the 3′-UT region between the nucleotide 19,682 and 19,885, closely to the last exon.

The sequence similarity between these elements and those used as reference (Lenstra et al., 1993; AC150707.3; GenBank: AC150561.6) ranges from 75 to 90%.

### Genotyping and Association of *CSN1S2* Polymorphisms With Milk Fatty Acid Composition Traits

To estimate the frequencies at the two polymorphic sites g.7539G>C and g.14067A>G, and to determine the possible haplotypes, specific genotyping protocols have been developed by the company Kbioscience (http://www.kbioscience.co.uk/genotyping/genotyping_intro.html).

The genotype distributions and the allelic frequencies of the two SNPs, determined in 747 buffaloes reared in Salerno, Caserta, and Potenza provinces (Italy) are reported in Table 2. The major alleles had a relative frequency of 0.83 and 0.51 for g.7539G and g.14067G, respectively, and the χ^2^ value showed that there was no evidence of departure from the Hardy–Weinberg equilibrium (*P* ≤ 0.05). Using Haploview software ver. 4.2 (http://www.broadinstitute.org/haploview/haploview), three different allelic combinations (out of the four expected) were observed: haplotypes 1 (7539G/14067A), 2 (7539G/14067G), and 3 (7539C/14067G). The first haplotype was the most represented with a frequency of 0.491, followed by the haplotypes 2 (0.336) and 3 (0.173). Although not observed, the fourth expected haplotype (7539C/14067A) was recorded on database (GenBank acc.no NC_037551.1).

**Table 2:** Genotyping data, allele frequency, relative frequencies of the SNP g.14067A>G at exons 16 and g.7539G>C in the splice donor site of intron 7 of the *CSN1S2* gene in the Mediterranean river buffalo population.

			**Genotype distribution**						**Allelic frequency**			
			**g.14067A>G**			**Obs**.	**Exp**.	**χ^**2**^**	**g.7539**		**g.14067**	
			**A/A**	**G/A**	**G/G**				**G**	**C**	**A**	**G**
Genotype distribution	g.7539G>C	G/G	192	229	94	515	512.1		0.83	0.17	0.49	0.51
		G/C	–	123	84	207	212.79	0.55				
		C/C	–	–	25	25	192.29					
	Obs.		192	352	203	747						
	Exp.		181.29	373.42	192.29							
	χ^2^		2.45									

The majority of mutations identified at this *locus* were either conservative (g.3165T>C, g.9220T>A, and g.11072C>T) or specific for an allele (g.9221A>G and g.14141C>G), and for these reasons, only the SNPs g.7539G>C and g.14067A>G were genotyped and used for running the model according to Cosenza et al. (2017a) (1).

Genotype distributions and allelic frequencies of both total- and sub-population genotyped are reported in Table 2 and Supplementary Table 3, respectively.

The analysis of the relationships between the *CSN1S2* polymorphisms and the FA profile showed a significant effect (*P* < 0.05) only for the SNP g.14067A>G on the content of palmitic acid in buffalo milk. In particular, the homozygous GG and heterozygous buffaloes showed a lower amount with 34.13% and 34.71% palmitic acid compared with the AA genotype (35.23%), respectively (Table 3).

**Table 3:** Least squares means of the SNP g.14067A>G genotypes for palmitic acid, estimation of average substitution effects (α) for the adenine to guanine replacement, and contribution of the polymorphism to the phenotypic variance (r^2^).

**SNP**	**Trait**	**P**	**Genotype**			**α**	**r^2^**
			AA (97)	AG (142)	GG (71)		
g.14067A>G	C16:0	0.05	35.23^a^	34.71^ab^	34.13^b^	0.55	0.15

a,b *Means within columns without a common superscript differ (P < 0.05)*.

## Discussion

Caseins αs1, β, αs2, and k have an important role for the production of milk-derived products in terms of quality and quantity. For this reason, in the last decades, many studies have been published in the main ruminant species (cow, sheep, and goat) about the identification of possible association between genetic markers and protein structure with milk traits of economic interest (Caroli et al., 2009; Selvaggi et al., 2014a,b; Ozdemir et al., 2018). Different from the abovementioned species, water buffalo has not been deeply investigated, and to our knowledge, the complete genomic sequence of the bubaline αs2-casein gene has not been reported yet. Therefore, this study focused first on the structure of the buffalo *CSN1S2* gene, exploring the genetic diversity within the Italian Mediterranean breed and testing possible associations between the detected polymorphisms and milk traits.

### Structure and Analysis of Mediterranean River Buffalo *CSN1S2* Gene

On the whole, the buffalo *CSN1S2* gene shares a similar organization with the bovine counterpart (Groenen et al., 1993), with some differences in intronic size, mainly as a consequence of the presence/absence of artiodactyla retroposons.

Transposable elements (TE) are the largest class of sequences in mammalian genomes, elements that replicate and jump throughout the genome in a manner similar to retroviruses. The TEs are distributed primarily as retrotransposons (98.62%) rather than transposons (1.38%). DNA transposons have been extensively studied beyond mammals (Berg and Howe, 1989; Capy et al., 1998; Craig, 2002), whereas they are not well-documented in mammalian genomes. Based on their size and mode of propagation, retrotransposons can be divided into two separate classes, the long terminal repeat (LTR) and non-LTR (Han, 2010). The non-LTR LINEs (long interspersed repeat elements, L1_Art, BovB) and SINEs (short interspersed repeat elements, BOV-A2, Bov-tA, tRNA, MIR, and others) are widely distributed and represent a major component of ruminant genomes. For example, the BovB LINEs and related SINEs occupy about 22% of the cow genome. In particular, two retroposon families, Bov-A2 and Bov-tA, are the most distributed in the genomes of ruminants (Lenstra et al., 1993). The Bov-A2 and Bov-tA retroposons share a common Bov-B LINE-derived region, called the Bov-A unit, suggesting a common origin for these two retroposons (Okada and Hamada, 1997; Shimamura et al., 1999).

Although many retroposons are common for the ruminants and non-ruminant species and, thus, are likely of ancestral origin, every species has a definite number of short interspersed nuclear elements, which contributes to make each genome specific for each species (Ramunno et al., 2004; Cosenza et al., 2005; Pauciullo et al., 2013, 2019).

The 13 repetitive elements observed at the buffalo *CSN1S2* gene and its promoter represent the 19.45% of the sequence deposited in the EMBL database. This figure decreases considerably in the bovine (GenBank no. M94327.1), caprine (GenBank no. NC_030813.1), and ovine (GenBank no. NC_040257.1) counterpart because of the presence/absence of other repetitive elements observed in these species. In particular, the bovine *CSN1S2* (similarity of 75.4 %) is characterized for the absence of the elements B and C and, at the same time, an expanded Bov-A2 (G element in buffalo in intron 12), which consisted of three Bov-A monomers (Bov-A3) in agreement with Onami et al. (2007). In sheep and goat, the number of retroposonic elements is lower. Both species have a similar gene structure (homology of 96%), and when compared to water buffalo, we noted the absence of elements C, G, I, and N. However, in the promoter region, there is an extra Bov-tA3, and in the intron 1, there is an expanded Bov-A2-derived sequences, which consisted of four Bov-A monomers: Bov-A4. Overall, it appears that the elements B, C, G, I, and N are rather young insertions. These ruminant-specific retrotransposon insertions are often polymorphic (present or absent) at orthologous *loci*, and they are highly informative genetic markers that can be considered a powerful phylogenetic tool for clustering studies, animal evolutionary history, population structure and demography, rather than the set-up of methods for the species discrimination in meat and dairy products (Cosenza et al., 2019).

The accumulation of interspersed repeats within or near genes has been studied in ruminants as well as in camelid casein genes (Groenen et al., 1993, Ramunno et al., 2004, Cosenza et al., 2009b, Pauciullo et al., 2013, 2014, 2019). It has not been observed that insertions within or near promoters or 3′ UTR can alter gene expression. Conversely, insertions into exons are often incorporated into existing protein-coding genes and modulate gene expression. For instance, the alleles E and G of the *CSN1S1* in goat and cattle, respectively, are characterized by the insertion of a truncated LINE in the last exon, which is, in both species, responsible for a reduction in transcriptional rate of the corresponding protein (Jansa Pérez et al., 1994; Rando et al., 1998). The interaction between the LINE sequence and the poly(A) sequence of the mature transcript, reduced the mRNA stability causing a rapid degradation of the transcript and a low protein synthesis efficiency (Rando et al., 1998). However, none of the elements observed at the *CSN1S2 locus* in the buffalo species would appear to be potentially responsible for differences in the gene expression.

Furthermore, these transposable elements are known to affect the genome in many other different ways: contributing to genome size increase, genomic instability, exonization, epigenetic regulation, RNA editing, and have the ability to generate microsatellites because they contain homopolymeric tracts and, in particular, mutations at many *loci* in the genome by Cordaux and Batzer (2009). In the buffalo, retroposons at the *CSN1S2 locus* are responsible for the majority of genetic variability. In fact, the comparison between the retroposonic sequences (4,157 and 4,139 bp for the alleles D and B, respectively) showed a homology level lower (98.92%) than that of the remaining part of the gene (99.49%) assessed on 16,164 and 16,179 bp, respectively, for alleles D and B. The increase in the genetic diversity of the retroposons is over eight-fold higher (8.23). Considering the number of mutational events (SNP, insertion/deletion) within each region, 24 mutations found in retroposons vs. 58 polymorphic sites found in the rest of the gene represent almost a double incidence of genetic variation (on average, one mutation every 160 vs. 279 bp, respectively). This finding confirms that interspersed repeats are major drivers of *CSN1S2* gene evolution.

The buffalo *CSN1S2* proximal promoter region showed, as expected, stronger similarities with sequences of other ruminants (about 96% with yak, cattle, zebu, and about 91–93% with goat, sheep, and common red deer) than those observed with non-ruminants (about 76% with lama, dromedary, pig, horse, donkey, and about 67% with rabbit).

### Detection of Genetic Variability and Allele Discovery

In the last decades, several studies have highlighted the importance of the genetic variability in non-coding regions, which regulates the expression of genes involved in milk quali-quantitative properties. Such polymorphisms are often located in the promoter region of milk protein genes that regulate their transcriptional rate and thus determine the amount of transcripts in milk (Malewski, 1998; Szymanowska et al., 2004a,b).

Also polymorphisms located in the 3′ untranslated region (UTR) are important because they could modify the target sequence of microRNA (miRNA), an important class of non-coding RNA responsible for the regulation of many physiological processes (including lactation) by influencing mRNA stability (Chen et al., 2010). So far, many SNPs located in non-coding regions of genes involved in the milk production traits have been identified. For instance, SNPs responsible of splicing mechanism modification (Cosenza et al., 2009a; Giambra et al., 2010; Balteanu et al., 2013) and mutations affecting transcription factor binding sites are associated with the regulation of gene expression (Kuss et al., 2003; Liefers et al., 2005; Ordovás et al., 2009; Pauciullo et al., 2012a,b; Yang et al., 2015; Cosenza et al., 2016, 2018b; Gu et al., 2019).

The comparison between the promoter sequences of alleles B and D at the *locus CSN1S2* of the water buffalo and the sequences recorded in GenBank has highlighted nine SNPs. Among them, only the mutation g.996T>A is located within the putative binding site for Oct-1, and consequently, it could affect the *CSN1S2* gene expression. The transcription factors Oct-1 belongs to a family of structurally related POU domain factors found throughout the eukaryotes. Oct-1 is the most studied member of the POU factors. It is expressed in all eukaryotic cells and regulates, either positively or negatively, the expression of a variety of genes (Dong and Zhao, 2007). In fact, mutations in the consensus sequences of the ubiquitous Oct-1 transcription factor are reported to reduce hormonal induction in different gene promoters, like the β-casein-encoding gene (*CSN2*) promoter in mice (Dong and Zhao, 2007) or the oxytocin gene (*OXT*) promoter in sheep (Cosenza et al., 2017b).

One of the main finding of this study was the discovery of a great genetic diversity at this *locus* and the understanding of phylogenetic relationship among the markers. Therefore, the clarification and rearrangement of allele nomenclature were considered a priority.

Regarding the high genetic variability found all over the *CSN1S2* gene, the most interesting polymorphisms identified are the transversion g.7539G>C at the donor splicing site of exon 7 (responsible for the *CSN1S2* B allele) and three SNPs in the coding region (g.11072C>T, g.12803A>T, and g.14067A>G) with two of them responsible for amino acid replacements.

Besides these SNPs, the comparative analysis with the bubaline *CSN1S2* sequences in GenBank identified further mutations. In total, eight observed markers allow to identify eight different alleles: *CSN1S2* A, B, B1, B2, C, D, E, and F (Table 1).

As a consequence, for the first time, it was possible also to propose an evolutionary pathway of the buffalo *CSN1S2* gene (Figure 1), as it was already published for different casein-encoding genes in ruminants (Formaggioni et al., 1999; Cosenza et al., 2008; Giambra and Erhardt, 2011).

Among the eight alleles, the *CSN1S2* C is of novel identification because it was never observed or reported earlier in databases. Furthermore, the identification of three B-derived alleles is interesting because they are characterized by the mutation g.7539G>C, which brings to the inactivation of the intron 7 splice donor site. In particular, *CSN1S2* B and B1 differ only for the conservative mutation g.11072C>T at the 18th nucleotide of exon 13, i.e., coding for the same protein 198-aa long vs. the 207 aa of the normal αs2-CN. Conversely, the haplotype of the allele *CSN1S2* B2 (DEL^58−66^ K^119^ g.11072C I^153^ T^181^ N^205^) likely suggests an interallelic recombination between the alleles D (K^128^ g.11072C I^162^ T^190^ N^214^) and B (DEL^58−66^ K^119^ g.11072T F^153^ A^181^ N^205^) or B1 (DEL^58−66^ K^119^ g.11072C F^153^ A^181^ N^205^) (Figure 1).

This hypothesis was strengthened by genomic sequencing data, the sequence of the *CSN1S2* B2 allele being available. Although a mutation-driven convergence cannot be excluded, an interallelic recombination/gene conversion event seems to be the most plausible. Indeed, a detailed comparative analysis at 94 polymorphic sites (15 belonging exclusively to allele B2) spanning a large part of the gene sequence (Supplementary Table 1) provides a haplotype formula allowing each allele to be precisely characterized. Thus, the B2 allele unequivocally appears to be a hybrid structure made of B-type allele sequences in its 5′ part (from the beginning of exon 12) followed by D allele sequences in its 3′ part (from exon 12 to 3′ flanking region). Following such a scheme, a recombination event would have occurred around exons 11 and 13. This is, to our knowledge, the first hypothesis of a genomic recombination event that happened for genetic polymorphism and generating a new allelic diversity at a *locus* encoding a milk protein in the buffalo. Similar examples were observed in the goat and llama for the *CSN1S1 locus* (Bevilacqua et al., 2002; Ramunno et al., 2005; Pauciullo et al., 2017). The resulting phylogenetic trees of the bubaline αs2-CN-encoding gene can certainly help to understand the history of buffalo breeds and their genetic distances, as recently illustrated also by Luo et al. (2020).

### Genetic Association With the Milk Palmitic Fatty Acid

The study of the correlations between the identified genetic variability and the phenotypic variability of animals is important especially for economic traits such as milk production and composition that are controlled by a cluster of genes (polygenes) where each gene has a small effect on the trait.

Different molecular genetic methods are used to identify the candidate genes involved in these quali-quantitative traits. Recently, a commercial buffalo SNP chip array, Axiom_Buffalo Genotyping Array 90K (Affymetrix), has been created to investigate the structure of buffalo populations (Iamartino et al., 2017) and performing genome-wide association studies (GWAS). However, the use of the array is very limited, and the few studies available still refer to bovine genome for the SNP positions and gene annotations. This represents a great restriction despite the recent efforts in the new annotation release of the buffalo genome (Low et al., 2019). For this reason, the genome annotation is still necessary in this species, as well as the understanding of the candidate gene functions and their mechanisms in the regulation of milk production traits. In this respect, the approach of candidate gene association study is still a powerful method in river buffalo, especially for markers falling within genes or regulatory sequences and with putative causative effects. Thus, an additional aim of the present study was the identification of possible associations between two genetic markers (g.7539G>C and g.14067A>G) found at this *locus* and the water buffalo milk traits.

In our study, the SNP g.14067A>G showed a significant association (P < 0.05) with the content of palmitic acid in buffalo milk.

Palmitic acid is the main SFA in milk fat in all investigate species (Markiewicz-Keszycka et al., 2013; Gantner et al., 2015). Palmitic acid, also known as palmitate and belonging to the class of organic compounds known as long-chain fatty acids (C16:0), exists in all living species, ranging from bacteria to humans, and it is found naturally in palm oil and palm kernel oil, as well as in meat, milk, butter, and cheese. Palmitic acid is an essential component of cell membranes, secretory and transport lipids, with crucial roles in protein palmitoylation and palmitoylated signal molecules (German, 2011). In milk, the C16:0 originates both from diet and endogenous synthesis by the mammary gland (Chilliard et al., 2007).

In buffalo raw milk, the percentage of palmitic acid is about 34.8% of the total SFA (Cosenza et al., 2017a). This percentage is the highest among those observed in the milk of the majority of ruminants such as cattle (31.6%), goat (23.1%), and sheep (19.8%), and non-ruminants such as donkey (20.9%) (Blasi et al., 2008) and camel (18.4%) (Gorban and Izzeldin, 1999). On the contrary, the contribution of the short-chain FA (C8:0, C10:0, and C12:0) is rather low compared with what was observed in other ruminant species (Correddu et al., 2017).

High concentrations of palmitic acid are also present in buffalo dairy products, such as mozzarella di Bufala Campana PDO (24.7%, Romano et al., 2008), yogurt (31.7 %, Naydenova et al., 2013), and ghee butter (28.7%, Peña-Serna et al., 2019).

Nutrition and supplementation of feed rations constitute a natural and economical way for farmers to increase the content of unsaturated fatty acids in milk (Chilliard et al., 2007), but some authors reported their negative effect on milk flavor (Stoop et al., 2008). Moreover, it can cause milk fat depression and decrease in milk yield (Markiewicz-Keszycka et al., 2013).

An alternative way of acting on the concentration of milk fatty acids is the application of genetic selection. Indeed, Stoop et al. (2008) found that there is a considerable genetic variation for fatty acid composition, with genetic variation being high for C16:0.

However, few studies have shown possible associations between genetic variability and the variability of palmitic acid concentration in milk. Schennink et al. (2007) found that the acyl CoA:diacylglycerol acyltransferase1 (*DGAT*1) 232A variant is associated with less C16:0 in cow milk. This association has also been observed by Bouwman et al. (2011), which suggests that the gene 1-acylglycerol-3-phosphate O-acyltransferase 6 (AGPAT6) might be a candidate for this association. Similarly, Zidi et al. (2010) detected a suggestive association between *PRLR* genotype and palmitic acid in goat.

Recently, many studies have been performed to identified possible associations with fatty acid composition in water buffalo milk (Misra et al., 2008; Pauciullo et al., 2010; Cosenza et al., 2017a, 2018a; Gu et al., 2017, 2019). In particular, Cosenza et al. (2017a) reported that the genotype CC at the oxytocin receptor (*OXTR*) was significantly associated to a lower level of palmitic acid in milk of Mediterranean river buffalo.

It is well-documented that palmitic acid is associated with obesity, with decreased insulin sensitivity that could increase risk of type 2 diabetes and higher cardiovascular disease risk through increased level of blood cholesterol much more of other SFAs (Mensink et al., 2003; Bermudez et al., 2014; Praagman et al., 2016; Imamura et al., 2018). Therefore, its presence at high concentrations in human diets has a negative impact on health, and it should be avoided preferring foods with higher concentrations of MUFA and/or PUFA. In this respect, increasing the frequency of *CSN1S2* GG and *OXTR* CC genotypes in river buffalo might guarantee a lower content of C16:0 in milk and dairy to be desirable for the consumer of buffalo products.

## Data Availability Statement

The datasets presented in this study can be found in online repositories. The names of the repository/repositories and accession number(s) can be found in the article/Supplementary Material.

## Ethics Statement

No animals were used in the present study. The samples used herein belonged to DNA collections available from past studies (Cosenza et al., 2017a, 2018a) already approved by different ethic committees. Therefore, according to the Committee on the Ethics of Animal Experiments of the University of Torino (D.R. n. 2128 released on 06/11/2015) further ethics approval was not required.

## Author Contributions

GC and AP conceived and designed the experiments. BA and DG performed the experiments. GC, AP, and GG analyzed the data. GC contributed reagents, materials, and analysis tools. GC and AP wrote the paper. GC, AP, GG, BA, and DG revised the article critically for important intellectual content. GC, AP, GG, BA, and DG gave final approval of the version to be published. All authors contributed to the article and approved the submitted version.

## Conflict of Interest

The authors declare that the research was conducted in the absence of any commercial or financial relationships that could be construed as a potential conflict of interest.
