# Supplementary material for: Complete CSN1S2 Characterization, Novel Allele Identification and Association With Milk Fatty Acid Composition in River Buffalo
Source: Front Genet. 2021 Feb 4;11:622494. doi: 10.3389/fgene.2020.622494 (PMC7890360; doi:10.3389/fgene.2020.622494)
Supplement: Supplementary file 1 [file Image_1.pdf]

```
                                          GR/PR    GR
gagaggttacaagatgtattagaaagctgttgataattatctaagcaatggaagataaactggcacagaaaaaatgtaggtagcatgtagttaccaactcactttatataaattatttct  120
----------------------------------------------------------------------------------------------------------c-------------  120
    POU1F1a
aattattttaatattGGCTTTCCTGGTGGCTCAGCGGGTAAAGAATCTGCCTGCAATGCGGGAGACCTGGATTTGATCCCTGGGTTGGGAAGAAACCCTGAAGGGAAAGGTTACCCATTC  240   A: Bov-tA2
-----------------------------------T------------------------------------------------------------------------------------  240

CAGTATTCTAGCCTGGAGAATTCATGGACTGTATAGTCAAGAGTCGGACATGACTGAACGACTTTa cttaagacttaagacaatattataaattggattaaatatagatattattattt  359
------------------------------------------------------------------a-----------------------------------------------------  360
                                  HNF-3beta   POU1F1a          GR                  C/EBP alpha Oct-1 POU1F1a  YY1
gacagattctgaaactaatgagaaaagttaaaaaagtaaatacttattaaaagttactgctagctagtaggagagctgagtttcccagtgtattacatagacttgcatttggctaacccta  479
-------------------------g----------------------------------------------------------------------------------------------  480
                                       POU1F1a                POU1F1a     C/EBP       Oct-1 POU1F1a
tttttagagttggcaaatgataagcagtgaataggaagaatgtatttttcacttattaatgaaattttaattaacatatccaaaatacaattttagatgcttatttaaaatggtcgtgac  599
------------------------------------------------------------------------------------------------------------------------  600
                               HNF-3beta                              Oct-1                      HNF-3beta  Oct-1
tagaagaggttctgcggtacagtctaggcaaaaaataaacaaaaaaagccttatgctgagcctcataactagtattaaaatgtggaagagctgagctacacaaacatgaaatatagataa  719
--------------t---------------------------------------------------------------------------------------------------------  720
                       AP-1          GR/PR    Oct-1                       YY1
tcatgacatattataaaccccacattacctagtatcatgttctaaatcaaactgagtagaagcattccaaggaatatggggtacaaaaattcgatagaaaagcaattctaatcatcatga  839
------------------------------g-----------------------------------------------------------------------------------------  840
  MGF     Oct-1                         Oct-1   YY1                TATA box
cttcttagaattcaaattctgttcaggtatttcaaaccacagaattaccatattatggaggaacaaggtataaatagtgttgtgccaatccatcagagATATTCCATTGCCTGGACTACT  959   Exon 1
------------------------------------------------------------------------------------------------------------------------  960
                    HNF-3beta POU1F1a Oct-1
TGTCTTCCTTTTAGGAAACGAGgtaatattttcatttatctttttgttattctatgtaccttgtaatattgtaaaacctaatagcattgttccaaaaagtatttttttttttagagaaaaa  1079
------------------------------------------------------------------------------------------------------------------------  1080

tgatatttattttcaatttatttggcttaaggtttccttcctTTATTTTATTTTTTTTATTTTTTTTTATATTCAAATTTTAAAGTTCTTTTTTTTTAATTTTATTTTATTTTTAAACTT  1199   B: L1_Art
------------------------------------------                 ----------------------------T-------   ----------------------  1179

TACATAACTGTATTAGTTTTGCCAAATATCAAAATGAATCTGCCACAGGTATACATGTGTTCCCCATCCTGAACCCTCCTCCCTCCTCCCTaatgtcagtgtaccttaagattctttgag  1319
--------------------------------------------------------------A---------------------------------------------------------  1299

aaattgtttgatagctaacatagctactgatttatctccctttaagtgcttcttaaaaaaattaacatccagatgctagaaattgttatactgctctatcttatgatgaaatgccacaat  1439
-------------a----------------------------------------------------------------------------------------------------------  1419

cttattttaaggatgagctaatcataatgaggtttttgaaaatccagggtgctgcacattagatgtgtgacctcctggatcactttcttcatctgttaatcagacacgtgtacctaacaa  1559
------------------------------------------------------------------------------------------------------------------------  1539

aattgttccacagactagagaaagaatttacaacatacctagtacaatgtctggcaatagcggatgcttcacaactaccactatttctatggttctcatttatcaacacaataaaggcat  1679
------------------------------------------c-----------------------------------------------------------------------------  1659
```

```
ttctttaattcttagttaatatttgtccatg         ctttcatccatggaaaataaaatatcttaagtaaaattgaggtaagtggctatatataatgaacttatttcaaacattta  1790
-------------------------------ctccagttt--------------------------------------------------------------------------------  1779

aattataaaatttaatatatttattatattttaatacattgtcattatgaaatgcttataaagtgaatgtcatgtgcattatcctgtaaaagaaaacaagaatatctgggattctttagt  1910
-----------------c------------------------------------------------------------------------------------------------------  1899

aggaatgataaattaataacaaaagcaggcaatgctaatctTTTTTTAATTTAATTTTATTTTATTTTTAAACTTTACAATATTGTATTAGTTTTGCCAAACATGGAAATGAATCCGCCA  2030   C: L1_Art
------------------------------------------------------------------------------------------------------------------------  2019

CAGGTATACCTGTGCTCCCCATCCTGAACCCTCCTCCCTCCTCCCTCCCTATACCCTCCCTCTGGGTCTTCCCAGTGCACCAGCCCCAAGCATCCAGTATCGTGCATctcttaagacaga  2150
----------C-------------------------------------------------------------------------------------------------------------  2139

gaataattcccatgcatatgcattctctaaatttgcacaggcaaagatcatcagcaaatttaacaattttgagtcaaataaaatcttgccgtttaaaaataattgatttcaaatttgtag  2270
------------------c-----------------------------------------------------------------------------------------------------  2259

atctacagagtaaaacactattatatgtcaaaaagtcactagaataactttatttcacttttcagttctttgtttatgcactggaaaagttgcattgatatgtagtcagcaaaccttggg  2390
------------------------------------------------------------------------------------------------------------------------  2379

gacttatatgtggtcatagtattagagattgagctggaggggatcttagaaatcaaatcttcttagttaaaagtcttgtttggtggttattccgtcaggttcagtgatgttcagtgactt  2510
---------------------------------------------------------c-----------c--------------------------------------------------  2499

gctcaaggttcagaagcctgtcccaggctaagctgggatcagcaggcttcttgtccccagcacagtgttattttcacctgccaattcttccccttatgagggaggcaggctccatgtttt  2630
------------------------------------------------------------------------------------------------------------------------  2619

ggctcacctgaaatctgattttaaaaagatttctgggtttttttgcatttctgatatgattctccataagatttaagaagaattgtaaaaaaaaatatggcatgttagtataaacatca  2750
--------------------------------------------------------------------------------------- --------------------------------  2738

ttatttttcatcactgtatctttatgtctggaatagtacctgacatagcaagtagtcattgaataagtgggtaaattaataaataatttagctatttatacatagggtcaattatgccac  2870
------------------------------------------------------------------------------------------------------------------------  2858

taatttggtatgcccaaatgagcctccacaatttagaaattaagattttacatttccttctccaggttttacattttgttgtgttaatttcttcttgtaaagaactcatcatattcaaat  2990
------------------------------------------------------------------------------------------------------------------------  2978

ccatgtgtttctcaatgaatctacttttatcagtcttcattgcccttttctaattatctgaagataatttaatacataattaatgaggaaatgtgtgattataaggagagtaaaactgtt  3110
------------------------------------------------------------------------------------------------------------------------  3098
                                         M  K  F  F  I  F  T  C  L  L  A  V  A  L  A  K  H
attaattagcttattgtctatctcacagGACCAAGTAAACATGAAGTTCTTCATTTTTACCTGCCTTTTGGCTGTTGCCCTTGCAAAGCATgtaagtataataagatacagatgagaata  3230   Exon 2
------------------------------------------------------------------------------------------------------------------------  3218

tattatgtaaatataagaatattaaattgtttatgaggataatgtcaggggtagacagcagggactatagaggaagtgtataaactttgaaacctataatcaaagatggccttggccctg  3350
------------------------------------------------------------------------------------------------------------------------  3338

ccttatgtaagttatgaagatggacaaaaataccacttctaaagtgttctccttctaggattggaaagatttttagttcgaagttaaaaagtacactttcttctgtaaacatttgaattt  3470
------------------------------------------------------------------------------t------t----------------------------------  3458
```

```
attggggaaatattttcacttcaagaatcacttcttaaaagtaagaaacatgaaaccataacaaggacttcttttaaagttcaggagagtcacagagaaaatgtagattcccaaaccatc  3590
------------------------------------------------------------------------------------------------------------------------  3578

tcaagcatatgtcagaatttcatcattcttgactccagtagctcagcaacaatgatcctttgataggaaacagggatgtgttgaatagtgctaactttaggagttGGGCTTCCCAGGTGG  3710   D: Bov-tA2
------------------------------------------------------------------------------------------------------------------------  3698

CTCAGTGGTAAAGAATCCACCTGCAATGCAGGAGACTCCAGTTCCATCCCTGGGTCAGGAAGATCCCATGGAGAAGGAAATGGCAACCAACTCCAGTATTCTTGCCTGGAGAAATCCTTG  3830
------------------------------------------------------------------------------------------------------------------------  3818

AACAGAAGAGCTTGGCAGGCTACAGGCCGTGAGTTGCAAAGAGTCAAACACAACTGAGAGACTAAATAGCAACAACtttgacttgccagataacctaatgcaaagtcaacccttcatttc  3950
------------------------------------------------------------------------------------------------------------------------  3938

tcaaagcaacatcttctagtgagaatgttacagaaatattcatatcatacaatgataaagaaggactagatgcttcacatgaagcgctttgaagatacaggcttgtaatctgttagctcc  4070
------------------------------------------------------------------------------------------------------------------------  4058

ctcaggagcaactgggcttcttcccttaccctgcagcctgtgctgccaacacctctcccatgcccagttctcactcagttcagaCTGGGCTTTTCTTGTGGCTCAGTTGCTAAAGAATCT  4190   E: Bov-tA2
------------------------------------------------------------------------------------------------------------------------  4178

GCCTGCAATGCTGGAGACCTGGGTTCAATACCAGGGTTGGGAAGATCCCCAAGACTAATCACTCCAGTATTCTGACCTGAAGAATTCCATGGACTACACAGTCCAGAGGGTCGCAGAGTC  4310
-------------------------------------------------------------------------------G--------------------------------A-------  4298

AGAtgctttcattcactttcacttttcagatgtaccgtatcatcagggtgaccagagttctttgctttctttccatttccacacctcacacatcaatcttgaaaagtttc  ttagtctc  4428
--------------------------------------------------------------------------------------------------------------tg--------  4418

taaagccaagccaagagcttagctacagtaagaattcctgttagatgaatataagataaaataaccttcagtggtcaattttacaccaagtttcattgtttcttatttataatctcctag  4548
-------------------------------------------------------g----------------------------------------------------------------  4538

tcttaattactcttctctgtaaatcagtaattgtcctcaatctttcccttaagagtaactaaatctagcaattaacaaaagctaaataaaacaaaatatcatggtattataaatatttta  4668
------------------------------------------------------------------------------------------------------------------------  4658

agattctgtagctcatgtgcacataaattatattttaaactgtgatagggtaaactaagaacccaacacttcctaaaagcaagaactacattaagctataaaaaggatttgtttgacata  4788
------------------------------------------------------------------------------------------------------------------------  4778

caaaatgatttgctggtaatattataggatataatattataataataatgaaaaaattcaactaaacatcatgcatttccagaaacaaattcctaaaagtctcttgccatcaaaacaaac  4908
------------------------------------------------------------------------------------------------------------------------  4898
                                                                                        T  M  E  H  V  S  S  S  E
aggaataatgataaaacaaaatagtgagataccttggcattttaaatctaccataaaactgagaactgtatttgcttttgtttatagACGATGGAACATGTCTCCTCCAGTGAGgtaaga  5028   Exon 3
------------------------------------------------------------------------------------------------------------------------  5018

tactttagtatcaatggaaaaccttatcagttagtaaaaacagaatatgacctaaactgttgtctttaaaataatttttctccagtgaatctagaactgtagaattagagaaaattggaa  5148
------------------------------------------------------------------------------------------------------------------------  5138
```

```
gtgcaaccataacgaaaaggtatatagagaggaataaaacccaaaaatcttcacattcaaatcaccaagattagattagtacatttttttctgaagttgtctcagtattaatgaggtatg  5268
------------------------------------------------------------------------------------------------------------------------  5258

ctcacaagtcatgatgaatattttgggagtacagtgacagggatccattctatgtttctgcaaaaaggactaaagctcttccggagccatattccatgctacttcctaaatgacttccca  5388
------------------------------------------------------------------------------------------------------------------------  5378

gctacaggtacataacaggttcccaccgtcacctccagtgttacctaagcattttcttgttttccggtgatgtgtttaatctctcttaaaaagacagaaaaaaaatgtgataataataat  5508
-----g------------------------------------------------------------------------------------------------------------------  5498

atgaaaaaaacactgaaaattgtacagaggaaatagaaaacctattctttcttccctgtggaagatacttggtaaagtttttgattcagatattcataacccaaagcacaatgcaaaatg  5628
------------------------------------------------------------------------------------------------------------------------  5618

agttaggtatatgaaatttaatagttatgtagagggagtgaaacatcagttaattttgttcggtaattcaggaaaagactaatatgttatctcctccatagataatgggtgtagatcgat  5748
-----------------a---------------c--------------------------------------------------------------------------------------  5738
                                                                 E  S  I  I  S  Q  E
atattattaagctaatatgaattcaatttaggtaaatgagtcatgttgtctttctttttctaagGAATCTATCATCTCCAGGAAgtaagtaccaaattctcaagtcaaatataatatat  5868   Exon 4
------------------------------------------------------------------------------------------------------------------------  5858
                                                             T  Y  K  Q  E  K  N  M  A  I  H  P  S  K
tttaactttctttatttaattaaagttataaaa       taaatttatttattttgtagACATATAAGCAGGAAAAGAATATGGCCATTCATCCCAGCAAGgtaaagttggattacttt  5981   Exon 5
--------------------c------------atttaaa----------------------------------------------------------------------t---------  5978

agactattaatctttcactgatgaaacagcttttctatcaaacatcgatctctttctcattcagtcaaaaagagaattatgattgtgattgtgaaaatggtgatgttttcattgttttgt  6101
----------------------------------------------a-------------------------------------------------------------------------  6098

ttaaatgctatctactaatgaaaggagttctgggaaaagatttgagagccatttttgagccacaatgttaaagcagaatatctttgaaagaatttcttgttaaagattcatcatctttac  6221
------------------------------------------------------------------------------------------------------------------------  6218

ttttcttccttaattctgtactcttttgttcacaaaatcatattttctttgctataactgtatgctcaagaatattacactacatttcaataaatgttatgatcttttggctttgttttc  6341
-----------------------------------------------------------------------------------------------------g-t----------------  6338
       E  N  L  C  S  T  F  C  K
ttttagGAGAACCTTTGCTCCACATTCTGCAAGgtatacactgatttcacatctgtagcataatgtgaagtaaaatattatagtattttaactacactgaaattaacctctatttggaaa  6461   Exon 6
------------------------------------------------------------------------------------------------------------------------  6458

atatatttcaaatcaatcaaacttgaaaaccaagattatctttaaacaatgagaccaaaattagtcttctcaattctatgactaaattaaatgaagaaaatatatctttgcatgaatcaa  6581
------------------------------------------------------------------------------------------------------------------------  6578

ctaacacatttgattcccagaatctatgaagaataaagaacttatcttttagtgtttttcagtatcaaacttattttatcctttttttaaaattctttttctgttgcaatattttcttaaa  6701
-------------------------------------------------------------c----------------------------------------------------t-----  6698

acagactcaatcaatttattatagaaacaatatatatttgcttcctaaagcatgtcagttggcctagtgatgtatttttcctaatacacaataagaaatgactattaaaatgagaaagaa  6821
--------------------------------------------------------------------------------------t---------------------------------  6818
```

```
aaacctatccatgccttgcctaaagttatcttgtatattcattcattcacttgacaaatcttttattcaaccagaactatgtccccaacattatcttagtctgtaggaatacaataataa  6941
------------------------------------------------------------------------------------------------------------------------  6938

ttatgagactcgctggggatacaacaataaacacagtaaacaaaaataaataccactgtcatcaaataggaaattcttttataacttatacaaaactcatacaatgaaaccgaatccagt  7061
------------------------------------------------------------------------------------------------------------------------  7058

                                                                                                             E  V  I  R
ctaatgcatctcttaatgagagagaaatttttccttttatattcaggagaaaatagcatagcaatgctgtggggaaatacatctaattatttttcttctctctttaagGAAGTTATAAGG  7181   Exon 7
-------------------------c----------------------------------------------------------------------------------------------  7178

 N  A  N  E  E
AATGCAAATGAAGAGgtgagaaaattttatacatttgaacatttttttgttcatattaccaatatgtaatattctaaaattagtaatggcatatgggaacatgtttaaatttccttttttta  7301
---------------c-------t------------------------------------------------------------------------------------------------  7298

gtaaactggaaaatgatttataagttgcttcaaaatgacagtttttccctgtccacttgagtaatgtccacctaaatcactgtgtattctcttgagccacttacatttaaatatatgatc  7421
------------------------------------------------------------------------------------------------------------------------  7418

atttttatctcttccacttatttgctaaaggtgattaaaaacaagcggccaagaagcctggttgcattgcttttatttcaatccttttaacttatcatgctgatgatcatgtttgtaata  7541
----------------------------------------------------------------------------------t-------------------------------------  7538

                                                                                                E  Y  S  I  G  S  S  S
agagacatatcccaagtaaacattaacatgcaaagaataatgtttgcatttcttctggtaaccagatttatattgcatatattatttttttcaagGAATATTCTATCGGCTCATCTAGTG  7661   Exon 8
---------------------------------------------------------------------------t--------------------------------------------  7658

E
AGgtaagagaccttttcttttaacattgaatagcaagttcatccaaaatagattttcagtagtgtgtagtaatttgttgttaacatcacaaatagggtgagcaatggagaaatcatttac  7781
------------------------------------------------------------------------------------------------------------------------  7778

ccaaacttaatgtcatagtggggagactggaaagtgactgtctacaccctggtgtacatgggtgttctttcacagagatatcaaatatctgatgccaaaggaaagaagtaaagacatgct  7901
------------------------------g------------------------------------------------------------------g----------------------  7898

ctggtattgatagaaatagccaagcaatgagcacaatggggaaaggtgtcggggaaagggagaaccatagccttggctcagagccttgaggcacagcctgaagacttcctacttaacatc  8021
--------------------------------------------------t---------------------------------------------------------------------  8018

taggaagcctgctttactgttgctctaggccacgggtctgtctggaatggagagccaatggcctcctacaaataccaatgtgtcatctagaaagggtatgctggattcagtttgcaacat  8141
---------------------t--------------------------------------------------------------------------------------------------  8138

ctcactaggccaactttgtaaccttgtttgtcacctgtctacactccttaagcatttgatattgaatgaataatgtagaattgatgaaaactttaaaattaaacatgttttcagtcacat  8261
------------------------------------------------------------------------------------------------------------------------  8258

gttattactttgaaactgaggactctgaattttcaatttattgaatctctaataaacaCTGTAACTCCAATATTTTGGCCACCTCATGCGAAGACTTGACTCATTGGGAAAGACCCTGAT  8381   F: Bov-B
------------------------------------------------------------------------------------------------------------------------  8378

ACTGGGAGGGACTGGGGGCAGGAGGAGAAGGGGACGACAGAGGATGAGATGGCTGGATGGCATCACCGATTCGATGGACATGAGTTTGGGTAAACTCCGGGAGTTGGTGATAGACAGGGA  8501
-------------------------------------------------------------------A---------------A------------------------------------  8498

GGCCTGGCGTGCTGCGATTCATGGGGTCGCAAAGAGTCGGACCCGACTGAGCTACTGAACTGAACTGAACTGAataaatactgtaatttctcagtagtagtagcttggattctaaatata  8621
------------------------------------------------------------------------------------------------------------------------  8618
```

```
                                                                                     E  S  A  E  V  A  T  E
cttaatgaattgacctttctactctagaacaatactaagactaaatcatgcaagttaagttaatcctcctttcttcccccgaagGAATCTGCTGAAGTTGCCACAGAGgtaactaatttt  8741   Exon 9
------------------------------------------------------------------------------------------------------------------------  8738

                                                                                     E  V  K  I  T  V  D  D  K  H  Y  Q
cattcaaataaaatgaaattcatatagcccagctttgttttcattttataaatttttataacaagtgttttgttttcttaacagGAAGTTAAGATTACTGTGGACGATAAGCACTACCAG  8861   Exon 10
-------------------------c----------------------------------------------------------------------------------------------  8858

 K  A  L
AAAGCACTGgtaaatttctcatacaaattataacttcaagtaaacagtcatcgtaaccactgcttcttctccagtgttcacataactattcatcccacttacagaaaattttatgttact  8981
------------------------------------------------------------------------------------------------------------------------  8978

aaacaacccttgttaggtaaatttgccatgttgtataaattatctaattaaataaagttctaagaaacttggttcttctataatctacttttgacacatagagaagattcaatactggag  9101
------------------------------------------------------------------------------------------------------------------------  9098

                              N  E  I  N  Q  F  Y  Q  K  F  P  Q  Y  L  Q  Y  L  Y  Q  G  P  I  V  L  N  P  W  D  Q  V
taaatattgataattttctttctctttagAATGAAATCAATCAGTTTTATCAGAAGTTCCCCCAGTATCTCCAGTATCTGTATCAAGGTCCAATTGTTTTGAACCCATGGGATCAGGTTA  9221   Exon 11
------------------------------------------------------------------------------------------------------------------------  9218

K  R  N  A  V  P  I  T  P  T  L
AGAGAAATGCTGTTCCCATTACTCCCACTCTGgtgagtgctgctttttatatgttgtttcttgtttatttgtatttctttttgtttttggtgagggatgagttcaggataaagatttgt  9341
------------------------------------------------------------------------------------------------------------------------  9338

aaaatgtctaaagataaaatgtcccaaagagacaagttttaacttagaaagtagaacaactgtaaaaatcttgtagtccagaaattaaatcacaaacaaaatatagctcaagtatagtaa  9461
-----------------------------------------------------------------c------------------------------------------------------  9458

caaataacaatagcaaccatagtgacaaataagtaatttaatttaaattgaacatacctgtcttaagagtatgcttctatatcattttgcttggcttctcaaacaattctctaagaggag  9581
------------------------------------------------------------------------------------------------------------------------  9578

atagtaatcattaatttcatgtaacaaaaagattgc ttgttaagaaaatataaagaagtgaagtaatttacctaggttccagg    ctaccatttctttcataaaacagtctgtctt  9696
------------------------------------t-----------------------------------------------ctct--------------------------------  9698

                                                                               N  R  E  Q  L  S  T  S  E
aaggaatgttttctcatatgaggaataaataatgaaagttttcatggtccttttttttttttttttttttggttatagAACAGAGAACAGCTCTCCACCAGTGAGgtaaggcatttacct  9816   Exon 12
--------------------------------------------------    ------------------------------------------------------------------  9814

acaaaggcaataatctgataactagtatgatattattgacttattatacactgtactagacagtctttgatgtagttgcttaagcaataaatctggatttacataaaaattgaaaaaatc  9936
------------------------------------------------------------------------------------------------------------------------  9934

tttccaattgaaaaactggaactttagatccctaagaaaaatctggggaaagcagaacaaaaatgtacacattgaaggatacaaagattggatctgtgctagtatactttgtcaaaatgc  10056
------------------------------------------------------------------------------------------------------------------------  10054

tgttactacttctcttttttataactcacttttaatattgctatcacaccatgagacctggtatagatcttaaattttagaaacagagtgggagaaatccaatgtattattctgaaataag  10176
------------------------------------------------------------------------------------------------------------------------  10174

aactcaaattaattcagatccatataaattgctgttatccaagtgtcttcctgcacatcacacataattaaacacattttcacatcacacaagaaattgcctagttttattcctacttca  10296
------------------------------------------  ----------------------------------------------------------------------------  10292
```

```
taatgaataaaatatttgagaaaagagaaataaaaatttcaTGCTGCTAAGTCACTTCAGTCATGTCTGACTCTGTGCGACCCCAGAGACAGCAGCCCACCAGGCTCCCCTGTCCCCGGG  10416   G: Bov-A2
-----------------------------------------------------------------------------------------------------------------G------  10412

ATTCTCCAGGCAAGAACACTGGAGTGGGTTGCCATTTCCTTCTCCAATGCATGAAAGTGAAAAGTGAAAGTGAAGTCGCTCAGTTGTGTCTGACTCCTAGCGACGCCATGGACTGTAGCC  10536
------------------------------------------------------------------------------------------------------------------------  10532

CACCAGGCCCCTCCGTCCATGGGATTTTCCAGGCAAGAGCACTGGAGTGGGGTGCCATCGCCTTTTCCGAAATTTCATAAGTAAGactaaaatgtatcatttcaattttttcttaaggta  10656
------------------------------------------------------------------------------------------------------------------------  10652

tctgccaactcctctcaggaagcaagtcaaaaccacttacattaaatacagttaagaaattactttttatgttgttagttataaaatattatttatttgcataaatataatcaggagaca  10770
------------------------------------------------------------------------------------------------------------------------  10778

atgtggttatgcataccagtcagataaaatgtatttacatatttattgattcattgatgaggtatgtcattcatcattcacttaagaaatatgcatttagccagcattatgccctttaca  10896
------------------------------------------------------------------------------------------------------------------------  10892

ttatgttagactcttgggatacaacagtgatttacaaagcaataaaaataaattgaattattctttataaaagaggagtttttctaaatatagtcaaaaggaaattatgtatagacaata  11016
------------------------------------------------------------------------------------------------------------------------  11012
                                      E  N  S  K  K  T  V  D  M
cagtgggaattaaatgtctaattcttttatttttaagGAAAATTCAAAGAAGACCGTTGACATGgtaagatattatttctatatgaagctattttaattaatattttcaaaatctaata  11136   Exon 13
------------------------------------------------------T-----------------------------------------------------------------  11132

tcaataattaggcttatctgaaacattctgcttttaataaaatagagttatatttgtaagttatttaaaaatattatttttctccagcacttaactctgttcctctcaattgtcttgaat  11256
------------------------------------------------------------------------------------------------------------------------  11252

tctttcaattaaggccttccatgtgaacaagacttttctatatcacaaaagttttatattatttccactgcttctttctttttttaatcaaattttactatttttgaactagatatttt  11376
------------------------------------------------------------------------------------------------------------------------  11372

acttcaattctttttttacctattatactattattctttttgtaaaatgagaaagaactaaaactccacaaatgttaaaattatactgctaaaaattcaatttcttggtccccaaattttg  11496
------------------------------------------------------------------------------------------------------------------------  11492

actattttttttatttctcccaaaatcttctaatagttcatccagtgagaaaatatatacttttaaaataaacaaccaactagtttgttgcacttttctgctgttaattattttaagcaca  11616
------------------------------------------------------------------------------------------------------------------------  11612

ttaggtatttgctaatacttaatttttttaacagaaccacatcaaaatagctaataaaataaagaaaaacacatgtaagaaaaatacacagattcagagtcattttagagatctgaaaggc  11736
------------------------------------------------------------------------------------------------------------------------  11732

agtatatccaaagggttgagtagctgttctagccatgctattgacaatgttcagctggaccctgattcattgataacaggcatttttcaaataagccaatgccaaataaacatcctgtca  11856
------------------------------------------------------------------------------------------------------------------------  11852

actgccttcaccatagttctaccctgaatctctctggttgtttaggggcttgaatactggattgggttaagggttgtgtaaatcacagttacaaaaacacttgctgtgttagaatgagtc  11976
------------------------------------------------------------------------------------------------------------------------  11972
```

```
tagtggaaaaacgcaatgggaagacatcccaggagaggatgaaacatggattgtggcccacagtcttatggcctgaggcacagtccaacatcaacctcattgagagctttaggagcatgc  12096
------------------------------------------------------------------------------------------------------------------------  12092

cttatcttttctttcaatttgggggtccatactcattaaaaAGGGCTTCCCAGATGGCGCTAGTGCTAAAGAACCAGCCTGCCAATGCAGGAGACAAGAAACACAGATTTGATCACTGGG  12216   H: Bov-tA1
------------------------------------------------------------------------------------------------------------------------  12212

TTGAGAAGATTGACTGAAGAAGGGCATGGCAACCCATTCCAGTATTCTTGCCTGGAGTATCCCATGGACAGAGGAGTCTGGCAGGCTATAGTCCATAGAGTTGCAAAGAGTCACATAGGA  12336
------------------------------------------------------------------------------------------------------------------------  12332

CTGAAGTGACTTAGCACGCAgggaggcacagccattaaaaaaacaatggtcttcccagaaaggatatgcaagctctgctacatacctcaagagcctgctttgttttagttttccctcttt  12456
------------------------------------------------------------------------------------------------------------------------  12452

ctctccctctctcctctactcccttgctttgcatctgctatactcgatgagaatttgacattatatgaaaaatatacgtaaatggaaactcaaaacatactttcagtcagtcagttgtca  12576
------------------------------------------------------------------------------------------------------------------------  12572

tatggacacagagaatgctctgataatgcatattcaaactcattaaattcataataaatttctataaaatagcactttgttacttgatagcagtgatttagccttgaattgttaaaaata  12696
------------------------------------------------------------------------------------------------------------------------  12692
                                                                                             E  S  T  E  V  I  T  K
attagctctagttctctactgtaatgatgcagacaaaattactggtgggctattcaagtaaagctgattttc ttttttttttttccataagGAATCAACAGAAGTAATCACTAAGgtaa  12815   Exon 14
------------------------------------------------------------------------t----------------------------------T------------  12812
                                                                                                            F
                                                                                        K  T  K  L  T  E  E  D  K  N  R
gtaatttctcttcaaaaaaaattaattacatgctatcctataatatttgcttttgtatttattgcagtttgtttttattattaacagAAAACTAAACTGACTGAAGAAGATAAGAATCGC  12935   Exon 15
----------------c-----------------------------------------------------a-------------------------------------------------  12932
 L  N  F  L
CTAAATTTTCTGgtaaattttacacaaacatttgagcacactaagattcagcttggacaccactgaaccacaatcctctcaacaacaacaaagcaaaacaaaaaaactcaca tttttt  13054
-----------------------------------------------------------------------------------------------------------------t------  13052

taaacaaattctaatttctataagtcttccttatactttatgaaaactattgcaaaaacattctcccattgtaccagctgtactattgccacgtatccaccagtcattctaaaaactcac  13174
------------------------------------------------------------------------------------------------------------------------  13172

tgacttttcattttgtaaaaataggaaattatcctaaagacagataccactgtgtttaatctctataataatctatagttatacccacataaatggttcaaaatagaacacagaggagag  13294
------------------------------------------------------------------------------------------------------------------------  13292

ggatatttaattacagaaTGCTGCTGCTGCTGTTAAATCACTTCAGTCAAGTCCAACTCTATGTGACCCCATAGACAGCAGCCCACCAGGCTCCCCCGTCCCTGGGATTCTCCAGGCAAG  13414   I: BOV-A2
--------------------------------C------G--------------------------------------------------------------------------------  13412

AACACTGGAGTGGGTTGCCATTTCCTTCTCCAATGCATGAAAGTGAAAAGTGAAAGTGAAGTCGCTCAGTCGTGTCCTACTCTAGCGATCCCATGGACTGCAGCCTACCAGGCTCCTCCG  13534
------------------------------------------------------------------------------------------------------------------------  13532

TCCATGGGATTTTCTAGGCAAAAGTACTGGAGTGGGGTGCCATTGCCTTCTCCgaattacagaataggtgacaggcaatatattatcatttacttctataaaataaatgacactcagaga  13654
------------------------------------------------------------------------------------------------------------------------  13652
```

```
ttaacaattggctcagtgtcgcagattctactgaggtaaatcacacattctatatgactctaaggactaaggtatttccacaaggtatggaaaagaagctgcaatctaagacactgttaa  13774
-----------------------------------t---------------------------------------------g--------------------------------------  13772

gaaattatcccctttccttcttacacaaatattttttttcttttttttcccacttaagcattttggcactagagattgaaataaccaaagaatatttttaaacctaccatttattttataaa  13894
------------------c-----------------------------------------------------------------------------------------------------  13892

agtaaacataattttatatgaataaactttactttgcttaagtctgtttggtatcatttagaatttatccaagtaaaactttatttggttaggtccaggtgttctgacacatgggataat  14014
------------------------------------------------------------------------------------------------------------------------  14012

                        K  K  I  S  Q  H  Y  Q  K  F  T  W  P  Q  Y  L  K  T  V  Y  Q  Y  Q  K  A  M  K  P  W  T  Q  P  K
ggaataattataattttcctagAAAAAAATCAGCCAGCATTACCAGAAATTCACCTGGCCCCAGTATCTCAAGACTGTTTATCAGTATCAGAAAGCTATGAAGCCATGGACTCAACCTAA  14134  Exon 16
----------------------------------------------------G-------------------------------------------------------------------  14132
  T  N  V  I  P  Y  V                               A
GACAAACGTTATTCCCTATGTGgtgagttctcccttttattttaattttaaaactgagttgtcttttgtaataaaaataagatagggaaatgaaatatggaaataaaattctaagttct  14254
------------------------------------------------------------------------------------------------------------------------  14252

aaagtaaaagctaaaacaaaattaaatgaatgttctaggctggaaattgaattataaatcatataggatgaatagagctacaataagcagaaataattagcatgatacattgtgcttgca  14374
------------------------------------------------------------------------------------------------------------------------  14372

ttggatttaaatttcataagtatttgcttatttcagttgattctatagcatttcctgaaggtgggtcagcatagaaaatattacttccatttcacaaaagtatctgaaattccaaaaagt  14494
------------------------------------------------------------------------------------------------------------------------  14492

agcctgaccaaactctaattaacatttctcctttgtttcatttttaaaaaatgtgactgaggaaatgtattttaacataaggaataagggagagagcaggaaattgaacctagagaacc  14614
------------------------------------------------------------------------------------------------------------------------  14612

aaaaatttaatataaaaatataagattggaatccagaagacctgacataaatgaacttttgatttggggcaagtcacttcatttatatggatttttttaatatgatttacttcattggat  14734
------------------------------------------------------------------------------------------------------------------------  14732

atacatatagcatttaaagatatcttccccatgattagagaaaataagaatttagtatctgtagtttgaatataatgtgccaaaatttttttctaaatatttccacagagttgtaaatatt  14854
------------------------------------------------------------------------------------------------------------------------  14852

                                                         R  Y  L  *
cattttgtttgttattataagcaattagctaaaacatgttacttctttctcttttaaagAGGTACCTTTAAGATTCTTGAATTAACTGCTTCTACCTGGTTATGgtaagtagctttagcc  14974  Exon 17
------------------------------------------------------------------------------------------------------------------------  14972

ttataatttaaacaataattgggatttttgtgtgaaggatgactaatattaagtaatatacatacgtgttaaattgCTTCAGTCATGCCTGACTCTGTGTGATCCTATCGACTATAGCCC  15094  L:Bov-tA1
------------------------------------------------------------------------------------------------------------------------  15092

ACCAGGCTCCTCTTCCATGGTATTCTCCAGGCAAGAATACTAGAGTGAGTTGCCATGCCCTCTTCCAAGGGATCTTCCTGATCTAGGGATCGAACCCATGTCTGTTACATCTCCTGCATT  15214
-----------------------------------------------G-----------------------------------C-------------------C----------------  15212

GGCAGGCAGGTTCTTACCACTAacgctgcatggaagtatatagcttcaactttaagtcatacttggtttatgaaatattttaaattttgcttatacctcctggattcacaaatgtaagtg  15334
-----------------------t------------------------------------------------------------------------t-----------------------  15332

atctgtatatttgcatgcattttataaatattttgaaaataaattatagagaaggttctctatagctgatgataaattcagtcagaagcaagagctctgggtggaaattgtcacaagcat  15454
------------------------------------------------------------------------------------------------------------------------  15452
```

```
aatggtcacagggatgttaacttgtcaccttagagctcagacccagaatttcagtttggtgaaagatatgcaatacagttttcgtacacaagttagtttaaaacaagcattattttctct  15574
------------------------------------------------------------------------------------------------------------------------  15572

ggaaaaaatcattattttttaaacacataaaacctacttctcctagtgattctctttgacattttaaacattgcctgaaaaccatgatgaacagtcaaggaaacaagtatacatcaatg  15694
------------------------------------------------------------------------------------------------------------------------  15692

ttgctttttgtgtagtaactttctctttgatattgtccttgcagtatataagtcatctccaatctcagcattctgtctcactcaagatttcttgttctttgtatatgttctgcaatagg  15814
------------------------------------------------------------------------------------------------------------------------  15812

acaaagtaggtttcagagaaaccagtagtaacagtggtagtaatttattggccttgcaatgcttgtcacaatcctaagccctttgcctatattaaatttcatgttcacaaaaacctACAT  15934    M:Bov-B
------------------------------------------------------------------------------------------------------------------------  15932

CAGATCAGATCAGTCGCTCAGTCATGTCCGACTCTTTGTGACCCCATGAATCGCATCACACCAGGCCTCCCTGTCCATCACCAACTCCCGGAGTTCACTCACACTCACGTCCATCGAGTC  16054
---------------A-------------C------------------------------------------------------------------------------------------  16052

AGTGATGCCATCCAGCCATCTCATCCTCTGGCGTCCCCTTCTCCTCCTGCCCCCAATCCCTCCCAGCATCAGAGTCTTTTCCAATGAGTCAACTCTTCACATCAGGTGGCCAAAGTACTG  16174
------------------------------------------------------------------------------------------------C-C---------------------  16172

GAGTTTCAGCTTGAGCATCATTCCCTCCAAAGAAATCCCAGGGCTGATCTCCTTCAGAATGGACTGGTTGGATCTCCTTGCAGTCCAAGGGACTCTCAAGAGTCTTCTCCAACACCACAG  16294
------------------------------------------------------------------------------------------------------------------------  16292

TTCAAAAGTATCAATTCTTCGGCGCTCAGCCTTCTTCACAGTCCAGCTCTCACATCCATACATGACCACAGGAAAAACCATTGCCTTGACTAGATGAACCTTTGGTGGCAAAGTAATGTC  16414
------------------------------------------------------------------------------------------------------------------------  16412

TCTACTTTTGAATATGCTATCTAGGTTGGTCATAACTTTCCTTCCAAGGAGTAAGCGTCTTTTAATTTTATGGCTGCAGTCACCATCTGTAGTGATTTTGGAACCCAGAAAAATAAAGTC  16534
------------------------------------------------------------------------------------------------------------------------  16532

TGACACTGTTTCCACTGTTTCCCCATCTATTTCCCATGAAGTGATAGGACCGGATGCCATGATCTTTGTTTTCTGAATGTTGAGCTTTAAGCCAACTTTTTCATTCTCCACTTTCACTTT  16654
------------------------------------------------------------------------------------------------------------------------  16652

CATCAAGAGGCTTTTGAGTTCCTCTTCACTTTCTGCCATAAGGGTGGTGTCATCTGCATATCTGAGGTTATTGATATTTCTCCCGGCAATCTTGATTCCAGTTTGTGTCTCTTCCAGTCC  16774
------------------------------------------------------------------------------------------------------------------------  16772

AGCGTTTCTCATGATGTACTCTGCATAGAAGTTAAATAAACAGGGTGACAATATACAGCCTTGACGAACTCCTTTTCCTATTTGGAACCAGGCTGTTGTTTCATGTCCAGTTCTAACTGT  16894
------------------------------------------------------------------------------------------------------------------------  16892

TGCTTCCTGACCTGCATACAAATTTCTCAAGAGGCAGACCAGGTGGTCTGGTAGTCCCATCTCTTTCAGAATTTTCCACAGTTTATTGTGATCCACACAGTCAAAGGCTTTGGCATAGCC  17014
------------------------------------------------------------------------------------------------------------------------  17012

AATAAAGCAGAAACAGATGTTTTTCTGGAACTCTCTTGGTTTTTCCATGATCCAGCAGATGTTGGCAATTTGATCTCTGGTTC  CTGCCTTTTCTAAAACCAGCTTGAACATCAGGAAG  17132
-----------------------------------------------------------------------------------CT-----------------------------------  17132
```

```
TTCACGGTTCACGTATTGCTGAAGCCTGGCTTGGAGAATTTTGAGCATTACTTTACTAGCGTGTGAGATGAGTGCGATTGTGTGGTAGTTTGAGCATTCTTTGGCATTGCCTTTCTTTGG  17252
---------------------------------------------------------------------------------------A--------------------------------  17252

GATTGGAATGAAAACTGACCTTTTCAGGTCCTGTGGCCACTGCTGAGTTTTCCAAATTTGCTGGCATATTGAGTGCAGCACTTTCACAGCATCATCTTTCAGGATTTGGAATAGCTCAAC  17372
------------------------------------------G---------------G-------------------------------------------------------------  17372

TGGAATTGCATCACCTCCCCTAGCTTTGTTCTTAGTGATGCTTCCTAAGGCCCACTTGACTTCACATTCCAGGATGTCTGGCTCTAGGTCAGTGATCACACCATCATGATTATctgggtc  17492
--------------------------------------------T--------------------------------------T---------------------G--------------  17492

atgaagatcttttttgttcagttgttctgtgtatcttgccatctcttcttaatatcttctgcttctattaggtccataccatttctgtcctttattgagctcatctttgcatgaaatgtt  17612
------------------------------------------------------------------------------------------------------------------------  17612

cctttggtatctctgattttcttgaagagatccctagtctttcccattctgttgttttcctctatttctttgcattgatcactgaagaaatattaaatttcatgttcacaaaaacctaca  17732
------------------------------------------------------------------------------------------------------------------------  17732

tagccaatattattagccacgttgagataaGGAAACTGCTGCTGCTGCTGCCAAGTTGCTTCAGTCGTGTCCGACTCTGTGCGACCCCATAAACGGCAGCCCACCAGGCTCCCCCATCCC  17852   N:BOV-A2
------------------------------------------------------------------------------------------------------------------------  17852

TGGGATTCTCCAGGCAAGAACACTGGAGTGGGTTGCCATTTCCTTCTCCAATGCATGAAAGGAAAAGTGAAAGTGAAGTCGCTCAGTCATGTCCGACTCTTCGCAACCCCATGGACTGCA  17972
------------------------------------------------------------------------------------------------------------------------  17972

GCCTACCAGGCTCCTCCGTCCATGGGATTTTCCAGGTTAGAGTACTGGAGTGGGGTGCCATTGCCTTCTCCTAAggaaactgagccccatggaaattaaataacttgttcatgatcacca  18092
------------------------------------------------------------------------------------------------------------------------  18092

tgtaaaagtggaaaagccaagaatccaacacagatttccaattctagtattcaaacactccactactgatgtcaagctacaacccaagtccaactaggggatttttttagaacacttttt  18212
------------------------------------------------------------------------------------------------------------------------  18212

tggctttaggttatgttgtcacaaacttgatttgctttgtcatatcattttttccctccatatgatgaaaaggatcagactacttcgcaaaataattttctgtgaagaaaaagaataact  18332
------------------------------------------------------------------------------------------------------------------------  18332

attatgaatatgagctgaatattcagttgtgtttaataaatagcctttttcactttgctataattatttatatacttattctgcatttaaaaaaaatgatcatgtgttccataatgtgag  18452
------------------------------------------------------------------------------------------------------------------------  18452

agacatgaaggagataaaaataaggtaccaaacttatcctcattaagctctattctagacaaagtcacagaaatgcaatgcaaggtgccagatgtaagttagagacatcagaaatatgtg  18572
------------------------------------------------------------------------------------------------------------------------  18572

gtagtacaaaaagatccagtgatgcaccaaacgtaagtcctaaagtaagtcctttcagataattagaaataggctaagcaaagcaatggcaaaggaaaaaaaaaaggtatcaaactgata  18692
------------------------------------------------------------------------------------------------------------------------  18692

gaaaaatgacaaaagaaacagaaaccaaaattgcaagtcagttttaaagaatttaatgattttaagcaatttcttaacgtatcagcccattgtcattttgcataatgattttgttttatt  18812
------------------------------------------------------------------------------------------------------------------------  18812

aaaatctagatttcaattaggttaaaaggttttaggtgcaggaagcaataacattttatgtttaataaattatttagaaaataagaataattaaatgttccttcaaagttgctgaagtgt  18932
------------------------------------------------------------------------------------------------------------------------  18932
```

```
aaatatttccactttataaataaatataaatattaatactacttttatttttggattaaagaaactggctatcagttttcactcatacattaaattatttcacaaatagttattaaaat  19052
------------------------------------------------------------------------------------------------------------------------  19052

cctaccatgtgctaggttatcatgttgaacactagaaaatattaataatagttacaaataatagcattttggatacatgtatatgcatggctgaatccctttgttgttcacttgaaacta  19172
------------------------------------------------------------------------------------------------------------------------  19172

ccacaacattgttaatcaaatatatcccaatacaaaataaattttaaagtttaaaaaaaataataacattctgctaaactagtatggatgagtatgtgtgggttctgtatacagacatac  19292
------------------------------------------------------------------------------------------------------------------------  19292

attatttcttgaatatagattgataggtaatgtagtattctgttttccttttcagGTTGGACTGGAAAATCTATCTTCTACAGTTTCACATCTACCACTTTTCCTCATACAACCAGAATG  19412    Exon 18
------------------------------------------------------------------------------------------------------------------------  19412

TTTGGAGTGGCACATCAATAAGACAAATCGCAGAGTATTTCCTGAATTATTTATACTCTCCCTGTTAGTTTATATTGAATATACTGGGTTTATATGGTGGTTTCATACAAACTTAGCTGA  19532
------------------------------------------------------------------------------------------------------------------------  19532
                                                            Polyadenylation site       G/T cluster
TGATTATTTAAAATCCTTTCCCTACTCTTTCTGAGTTATAGAAATATTTTTCTTTCCCTCTGAAAATAAAATTTCAAGTGCattctttggtgtgtaatcatcatttccatcgtcatactg  19652
------------------------------------------------------------------------------------------------------------------------  19652

gcccatgaggctgtggattataatattgtTTGTTGTTTAGTTGCTAAGTCATGTTCAACTCTTTGCAACCCCATGACCCACCAGGCTCCTCTGTCCATGGGATTTCCCAGGCAAGAATAC  19772    O:Bov-tA2
------------------------------------------------------------------------------------------------------------------------  19772

CGGAGTAGGTAACCATTTCCTTCTCCAGGGGATCTTTCTGACCCAGGGATTAAACCCTGGTCTCCTGAATTGCAGGTAAATTCTTTTCTGCTGAGCCACCGGGGAAGGCTTCAtggatta  19892
------------------------------------------------------------------------------------------------------------------------  19892

taatataaagagtcttataatataaaagagtcctatcagtaactctgaaccagcttgactaccatcaaaagaaccatacactttctgagtaattcaatcttatttctcataaatattata  20012
------------------------------------------------------------------------------------------------------------------------  20012

ttctccaaccacagctaggtttacagtattattaaatcatcagtctaccttttttttttttttttactttgtgctcattatctgccatagcattaaccccaaatgttcaccagcggctt  20132
-------------------------------------------------    -------------------------------------------------------------------  20128

cagtcctcctgtctctggtcaccttatcacactaaagaagggagtcaggcattggaaacataaatcttccaaagccccctattaatggtattaacaactggagaaatagcaaaaacaaat  20252
------------------------------------------------------------------------------------------------------------------------  20248

gagttcatgcatggaacataagaaaactctagaagggtgacatatccaaagaagacagagagataggaa  20321 CSN1S2 D
---------------------------------------------------------------------  20317 CSN1S2 B
```

Figure S1. Homology between the nucleotide (nt) sequences of the whole Mediterranean river buffalo *CSN1S2* D (GeneBank acc. no. MW159135) and B (GeneBank acc. no. MW159136) alleles plus parzial 5' and 3' flanking region. Dashes indicate nucleotides identical between the two sequences. Peptide leader encoding region, microsatellites sequences, polyadenylation site and G/T cluster are underlined. The stop codon is symbolized by *. Congruent and putative factors are underlined or boxed. Exons sequences are in uppercase and shaded bold letters and Artiodactyla retroposon sequences are in uppercase and italics. Alignment was performed using DNAsis pro Software v2.0 (Hitachi) in combination with manual adjustment by eye.
