# Supplementary material for: Complete CSN1S2 Characterization, Novel Allele Identification and Association With Milk Fatty Acid Composition in River Buffalo
Source: Front Genet. 2021 Feb 4;11:622494. doi: 10.3389/fgene.2020.622494 (PMC7890360; doi:10.3389/fgene.2020.622494)
Supplement: Supplementary file 2 [file Table_1.doc]

| **Description** | **Primers sequence (5’-3’)** | | **Ta** |
| --- | --- | --- | --- |
| Exon 1 and flanking regions | Forward | GAGAGGTTACAAGATGTATT | 54°C |
|  | Reverse | CATAAGATAGAGCAGTATAAC |  |
| Intron 1 | Forward | GTGTACCTTAAGATTCTTTGA | 55°C |
|  | Reverse | TTCTGAACCTTGAGCAAG |  |
| Exon 2 and flanking regions | Forward | ATGTGGTCATAGTATTAGAG | 54°C |
|  | Reverse | TGGAGTCAAGAATGATGAA |  |
| Intron 2 | Forward | CACTTCAAGAATCACTTCTTA | 54°C |
|  | Reverse | ACATGTTCCATCGTCTATA |  |
| Exons 3 and 4 and flanking regions | Forward | GTCTCTTGCCATCAAAAC | 57.4°C |
|  | Reverse | TGGCCATATTCTTTTCCTGCTT |  |
| Exons 5, 6 and 7 and flanking regions | Forward | GGAAGTAAGTACCAAATTCT | 54°C |
|  | Reverse | CCCATATGCCATTACTAATT |  |
| Exons 7 and 8 and flanking regions | Forward | CCGAATCCAGTCTAATGCA | 54°C |
|  | Reverse | GTCACCTGTCTACACTCCT |  |
| Exons 8, 9 and 10 and flanking regions | Forward | CAGTTTTATCAGAAGTTCCC | 55°C |
|  | Reverse | GGGAACTTCTGATAAAACTG |  |
| Exons 11 and 12 and flanking regions | Forward | TGTTAGGTAAATTTGCCATG | 54°C |
|  | Reverse | GGATAACAGCAATTTATATGG |  |
| Exon 12 and flanking regions | Forward | GACCTGGTATAGATCTTAAA | 54°C |
|  | Reverse | TCTAAAATGACTCTGAATCTG |  |
| Intron 13 | Forward | CAGATTCAGAGTCATTTTAGA | 54°C |
|  | Reverse | TATCCTTTCTGGGAAGAC |  |
| Exons 14 and 15 and flanking regions | Forward | GTCTTCCCAGAAAGGATA | 55°C |
|  | Reverse | TTGTTGAGAGGATTGTGG |  |
| Exons 15 and 16 and flanking regions | Forward | CACTAAGGTAAGTAATTTCTCT | 55°C |
|  | Reverse | CCATATTTCATTTCCCTATCT |  |
| Exons 16 and 18 and flanking regions | Forward | AGAAAAAAATCAGCCAGCAT | 56°C |
|  | Reverse | TCCAGGAGGTATAAGCAAAA |  |
| Partial intron 17 | Forward | TCAACTTTAAGTCATACTTGG | 55°C |
|  | Reverse | AGGGATCTCTTCAAGAAAAT |  |
| Exon 18 and flanking regions | Forward | TTCTATTAGGTCCATACCA | 55°C |
|  | Reverse | ATTATAATCCACAGCCTCA |  |
| 3’UTR | Forward | TTTCAAGTGCATTCTTTGG | 55°C |
|  | Reverse | GAAGACAGAGAGATAGGAA |  |

Table S1. Primers and annealing temperatures (Ta) used for amplification of the buffalo *CSN1S2* gene. Numbering is according to the sequence of the bubaline genome (GenBank accession no. NC_037551.1) and bubaline mRNA sequence (GenBank accession nos. FM865618.1, FM865619.1).
