# Supplementary material for: Complete CSN1S2 Characterization, Novel Allele Identification and Association With Milk Fatty Acid Composition in River Buffalo
Source: Front Genet. 2021 Feb 4;11:622494. doi: 10.3389/fgene.2020.622494 (PMC7890360; doi:10.3389/fgene.2020.622494)
Supplement: Supplementary file 3 [file Table_2.doc]

| **Description*** | ***CSN1S2* D (GeneBank MW159135)** | | ***CSN1S2* B (GeneBank MW159136)** | | ***CSN1S2* B2 (GeneBank NC_037551.1**)** | |
| --- | --- | --- | --- | --- | --- | --- |
|  | **Mutation** | **Location** | **Mutation** | **Location** | **Mutation** | **Location** |
| 5’ region | T | 107 | C | 107 | T | 32040231 |
|  | G | 156 | T | 156 | T | 32040182 |
|  | – | 306-307 | Ins A | 307 | Ins A | 32040031 |
|  | A | 385 | G | 386 | G | 32039952 |
|  | C | 614 | T | 615 | T | 32039723 |
|  | A | 749 | G | 750 | A | 32039497 |
| Intron 1 | TTATTTTATTTTTTTTA | 1122-1138 | Del TTATTTTATTTTTTTTA | 1122-1123 | TTATTTTATTTTTTATA | 32039215-32039199 |
|  | C | 1167 | T | 1151 | C | 32039170 |
|  | TTAA | 1175-1178 | Del TTAA | 1158-1159 | Del TA | 32039162-32039161 |
|  | C | 1262 | A | 1242 | A | 32039079 |
|  | G | 1333 | A | 1313 | G | 32039006 |
|  | CCC | 1357-1359 | CCC | 1337-1339 | Del C (CC) | 32039020-32039019 |
|  | T | 1602 | C | 1582 | T | 32038738 |
|  | – | 1710-1711 | Ins CTCCAGTTT | 1691-1699 | Ins CTCCAGTTT | 32038630- 32038640 |
|  | T | 1808 | C | 1797 | C | 32038523 |
|  | T | 2041 | C | 2030 | C | 32038290 |
|  | T | 2169 | C | 2158 | C | 32038162 |
|  | T | 2448 | C | 2437 | T | 32037883 |
|  | A | 2460 | C | 2449 | C | 32037871 |
|  | GG | 2631-2631 | GG | 2620-2621 | Del G (G) | 32037700 |
|  | TTTTTTTT | 2668-2675 | TTTTTTTT | 2657-2664 | Ins T (TTTTTTTTT) | 32037663-32037655 |
|  | AAAAAAAAA | 2718-2726 | Del A (AAAAAAAA) | 2707-2714 | Del A (AAAAAAAA) | 32037612-32037605 |
| Intron 2 | C | 3429 | T | 3417 | T | 32036902 |
|  | A | 3436 | T | 3424 | A | 32036895 |
|  | C | 4237 | C | 4225 | T | 32036094 |
|  | A | 4270 | G | 4258 | G | 32036061 |
|  | G | 4303 | A | 4291 | A | 32036028 |
|  | – | 4420-4421 | Ins TG | 4409-4410 | Ins TG | 32035910-32035909 |
|  | A | 4484 | G | 4474 | G | 32035845 |
|  | T | 4857 | T | 4847 | C | 32035472 |
| Intron 3 | A | 5394 | G | 5384 | A | 32034935 |
|  | T | 5646 | A | 5636 | A | 32034941 |
|  | G | 5662 | C | 5652 | C | 32034667 |
| Intron 4 | T | 5889 | C | 5879 | C | 32034440 |
|  | – | 5905-5906 | Ins ATTTAAA | 5896-5902 | - | 32034429-32034428 |
| Intron 5 | G | 5972 | T | 5969 | T | 32034357 |
|  | G | 6028 | A | 6025 | A | 32034301 |
|  | A | 6323 | G | 6320 | T | 32034006 |
|  | C | 6325 | T | 6322 | C | 32034004 |
| Intron 6 | G | 6643 | C | 6640 | C | 32033686 |
|  | – |  | – |  | Ins G | 32033685 |
|  | C | 6696 | T | 6693 | C | 32033632 |
|  | C | 6788 | T | 6785 | T | 32033540 |
|  | A | 7087 | C | 7084 | A | 32033386 |
| Intron 7 | G | 7197 | C | 7194 | C | 32033131 |
|  | A | 7205 | T | 7202 | T | 32033123 |
|  | C | 7504 | T | 7501 | T | 32032824 |
|  | C | 7617 | T | 7614 | T | 32032711 |
| Intron 8 | A | 7812 | G | 7809 | G | 32032516 |
|  | A | 7879 | G | 7876 | G | 32032449 |
|  | G | 7952 | T | 7948 | T | 32032376 |
|  | G | 8043 | T | 8040 | T | 32032285 |
|  | G | 8449 | A | 8446 | A | 32031879 |
|  | G | 8465 | A | 8462 | G | 32031863 |
| Intron 9 | A | 8767 | C | 8764 | C | 32031562 |
| Intron 11 | A | 9407 | C | 9404 | C | 32030921 |
|  | TT | 9618-9619 | Ins T (TTT) | 9615-9617 | Ins T (TTT) | 32030710- 32030708 |
|  | CT | 9665-9666 | Ins CTCT (CTCTCT) | 9663-9668 | Ins TTCTCT | 32030655-32030650 |
|  | TTTTTTTTTTTTTTTTTTTT | 9747-9766 | Del TTTT (TTTTTTTTTTTTTTTT) | 9749-9764 | Del TT  (TTTTTTTTTTTTTTTTTT) | 32030574-32030557 |
| Intron 12 | GTGT | 10219-10222 | Del GT (GT) | 10217-10218 | GTGT | 32030104-32030101 |
|  | C | 10410 | G | 10406 | G | 32029913 |
| **Exon 13** | **C** | **11072** | **T** | **11068** | **C** | **32029251** |
| Intron 13 | A | 11922 | A | 11926 | T | 32028397 |
|  | TTTTTTTTTTTT | 12769-12780 | Ins T (TTTTTTTTTTTTT) | 12765-12777 | TTTTTTTTTTTT | 32027554-32027543 |
| **Exon 14** | **A** | **12803** | **T** | **12800** | **A** | **32027520** |
| Intron 14 | A | 12832 | C | 12829 | A | 32027491 |
|  | G | 12886 | A | 12883 | A | 32027437 |
| Intron 15 | TTTTTTT | 13049-13055 | Ins T (TTTTTTTT) | 13046-13053 | TTTTTTT | 32027274- 32027268 |
|  | T | 13327 | C | 13325 | C | 32026996 |
|  | A | 13334 | G | 13332 | A | 32026989 |
|  | G | 13690 | T | 13688 | G | 32026633 |
|  | A | 13736 | G | 13734 | A | 32026587 |
|  | T | 13793 | C | 13791 | T | 32026530 |
| **Exon 16** | **R** | **14067** | **G** | **14065** | **G** | **32026256** |
| Intron 16 | T | 14499 | T | 14497 | C | 32025824 |
|  | C | 14517 | C | 14515 | T | 32025806 |
|  | C | 14896 | C | 14894 | T | 32025427 |
| Intron 17 | A | 15142 | G | 15140 | A | 32025181 |
|  | T | 15178 | C | 15176 | T | 32025145 |
|  | G | 15198 | C | 15196 | G | 32025125 |
|  | C | 15238 | T | 15236 | C | 32025085 |
|  | C | 15311 | T | 15309 | C | 32025012 |
|  | G | 15950 | A | 15948 | G | 32024373 |
|  | G | 15964 | C | 15962 | G | 32024359 |
|  | T | 16151 | C | 16149 | T | 32024172 |
|  | A | 16153 | C | 16151 | A | 32024170 |
|  | CT | 17098-17099 | Ins CT (CTCT) | 17096-17099 | Ins CT (CTCT) | 32023225-32023224 |
|  | A | 17220 | G | 17220 | A | 32023101 |
|  | C | 17295 | G | 17295 | C | 32023026 |
|  | T | 17311 | G | 17311 | T | 32023010 |
|  | C | 17417 | T | 17417 | C | 32022904 |
|  | C | 17456 | T | 17456 | C | 32022865 |
|  | A | 17478 | G | 17478 | A | 32022842 |
| 3’ region | TTTTTTTTTTTTTTTTT | 20062-20078 | Del TTTT (TTTTTTTTTTTTT) | 20062-20074 | TTTTTTTTTTTTTTTTT | 32020259-32020243 |

**Table S2.** Polymorphisms detected at Mediterranean river buffalo *CSN1S2* *locus* by the comparison of the genomic sequences of D, B and B2 alleles. * The comparison is relative to the *CSN1S2* D allele. ** From 32020000 to 32040337 complement. Exonic mutation are indicated in bold.
