# Supplementary material for: Complete CSN1S2 Characterization, Novel Allele Identification and Association With Milk Fatty Acid Composition in River Buffalo
Source: Front Genet. 2021 Feb 4;11:622494. doi: 10.3389/fgene.2020.622494 (PMC7890360; doi:10.3389/fgene.2020.622494)
Supplement: Supplementary file 4 [file Table_3.doc]

|  | **Genotypic frequency** | | | | | | **Total** | **Allelic frequency** | | | | **Relative frequencies used in the model** | | | | **Farms (n)** |
| --- | --- | --- | --- | --- | --- | --- | --- | --- | --- | --- | --- | --- | --- | --- | --- | --- |
|  |  |  |  |  |  |  |  |  |  |  |  | g.7539G>C | | g.14067A>G | |  |
|  | g.7539G>C | | | g.14067A>G | | |  | g.7539 | | g.14067 | | Genotype | Cow (n) | Genotype | Cow (n) | 14 |
|  | G/G | G/C | C/C | A/A | G/A | G/G |  | G | C | A | G | G/G | 68.71 | A/A | 31.29 |  |
| **Obs.** | 213 | 88 | 9 | 97 | 142 | 71 | 310 | 0.83 | 0.17 | 0.54 | 0.46 | G/C | 28.39 | G/A | 45.81 |  |
| **Exp.** | 213.06 | 87.88 | 9.06 | 91.04 | 153.90 | 65.04 |  |  |  |  |  | C/C | 2.90 | G/G | 22.90 |  |
|  | χ^2^= 0.0006 | | | χ^2^= 1.8562 | | |  |  |  |  |  | **Total** | 100 |  | 100 |  |

**Table S3**. Genotyping data, allele frequency, Hardy–Weinberg equilibrium (χ^2 -^ P≤0.05) and relative frequencies for the SNPs g.7539G>C and g.14067A>G at *CSN1S2 locus* in the Mediterranean river buffalo sub population considered in running the model.
